# USP11 deubiquitinates RAE1 and plays a key role in bipolar spindle formation

**DOI:** 10.1371/journal.pone.0190513

**Published:** 2018-01-02

**Authors:** Anna Stockum, Ambrosius P. Snijders, Goedele N. Maertens

**Affiliations:** 1 Imperial College London, Department of Medicine, Division of Infectious Diseases, Norfolk Place, London, United Kingdom; 2 Francis Crick Institute, The Crick Mass Spectrometry Science Technology Platform, 1 Midland Road, London, United Kingdom; Virginia Tech, UNITED STATES

## Abstract

Correct segregation of the mitotic chromosomes into daughter cells is a highly regulated process critical to safeguard genome stability. During M phase the spindle assembly checkpoint (SAC) ensures that all kinetochores are correctly attached before its inactivation allows progression into anaphase. Upon SAC inactivation, the anaphase promoting complex/cyclosome (APC/C) E3 ligase ubiquitinates and targets cyclin B and securin for proteasomal degradation. Here, we describe the identification of Ribonucleic Acid Export protein 1 (RAE1), a protein previously shown to be involved in SAC regulation and bipolar spindle formation, as a novel substrate of the deubiquitinating enzyme (DUB) Ubiquitin Specific Protease 11 (USP11). Lentiviral knock-down of USP11 or RAE1 in U2OS cells drastically reduces cell proliferation and increases multipolar spindle formation. We show that USP11 is associated with the mitotic spindle, does not regulate SAC inactivation, but controls ubiquitination of RAE1 at the mitotic spindle, hereby functionally modulating its interaction with Nuclear Mitotic Apparatus protein (NuMA).

## Introduction

Ubiquitination, the post-translational modification that results in the addition of minimally one ubiquitin molecule to a protein, regulates a wide range of cellular processes [1]. Whilst the addition of ubiquitin chains is a fine-tuned process, the removal by DUBs is equally well organized. Deubiquitination is catalysed by several families of DUBs of which the Ubiquitin Specific Proteases (USPs) are the largest [2]. USP11 was originally identified by Valle and colleagues [3] and because of its role in a plethora of cellular processes, including DNA damage response [4–6], modulation of cellular senescence [7] and intracellular signaling [8–11], USP11 is an interesting target for cancer therapy.

With the goal of identifying novel binding partners and/or substrates of USP11, we performed a proteomics screen using Flag-tagged USP11 as bait and identified RAE1 as a substrate of USP11. Originally identified as an mRNA export protein [12–14], more recent work has shown that RAE1, in complex with Nucleoporin 98 (NUP98), also contributes to mitotic checkpoint regulation [15–18]. The APC/C is activated by binding to either CDH1 or CDC20, a process that is regulated by a number of phosphorylation events [19]. During SAC, association of the RAE1:NUP98 complex with CDH1 inhibits APC/C^CDH1^ [15, 17, 18]. Likewise, CDC20 is bound by the mitotic checkpoint complex which prevents activation of APC/C by CDC20 [20, 21]. Once all the kinetochores have been attached to the spindle microtubules and chromosomes are aligned at the metaphase plate, the SAC is inactivated, CDH1 and CDC20 are released from their respective complexes and can now activate the APC/C, which results in securin and cyclin B degradation allowing metaphase to anaphase progression [15, 17, 18]. More recently, RAE1 was reported to play a role in bipolar spindle formation [22–25]. NuMA, a 237 kDa protein, is associated with microtubules during mitosis and harbours a highly conserved Gle2-binding site (GLEBS) motif that is recognized by RAE1 [25]. The balance of NuMA and RAE1 protein levels in the cell is critical to bipolar spindle formation, since knock-down or over-expression of either protein alone results in abnormal spindle formation, whilst ablation or ectopic expression of both proteins can restore the wild type phenotype [22, 25, 26].

We show here that deubiquitination of RAE1 by USP11 does not regulate RAE1 protein levels nor the SAC. Instead, USP11 is a key player in proper mitotic spindle formation. As previously shown for RAE1, USP11 knock-down can restore bipolar spindle formation in cells with reduced NuMA expression. Finally, we show that USP11 regulates a ubiquitinated form of RAE1 at the mitotic spindle, likely modulating its functional interaction with NuMA.

## Results

### USP11 interacts with RAE1, SPRYD3, KCTD6 and PAM

**Fig 1:**
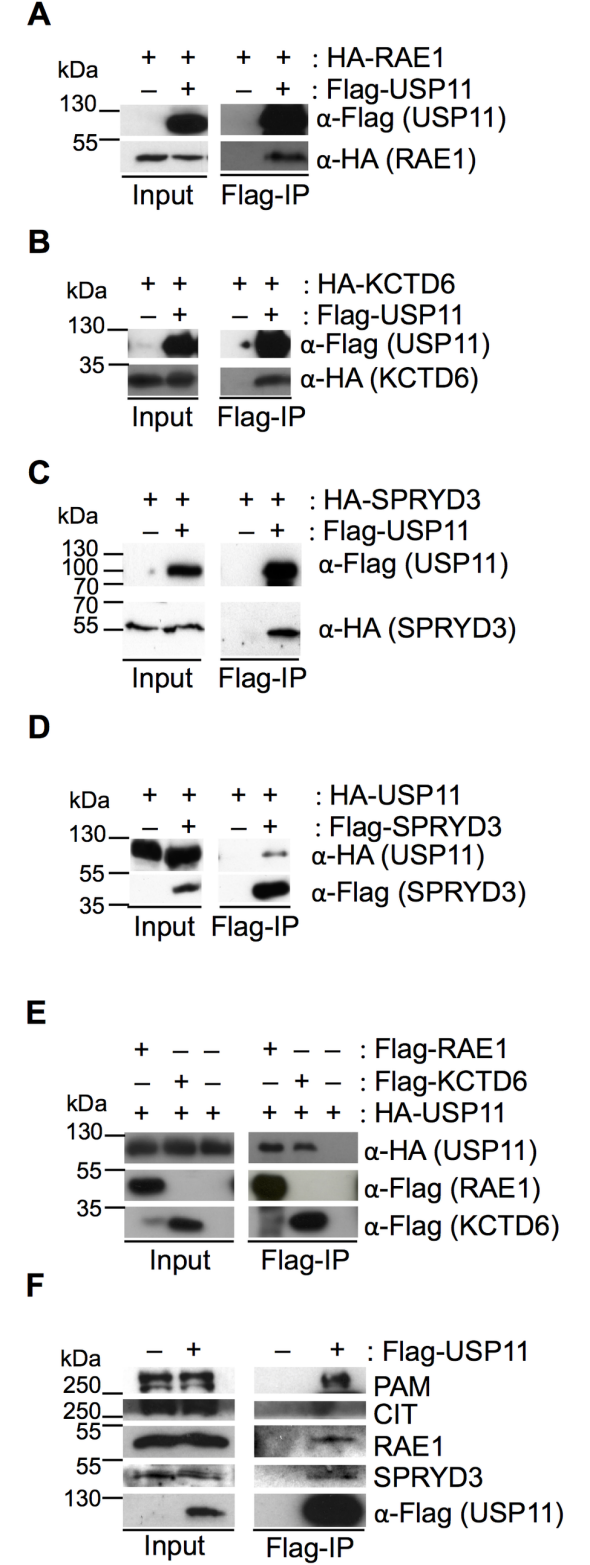
USP11 interacts with RAE1, KCTD6, SPRYD3 and PAM. 293T cells were transfected with plasmids expressing the indicated Flag- or HA-tagged fusion proteins. “–” refers to empty plasmid control. Input and Flag-IP samples are indicated underneath each panel. Antibodies and fusion proteins are indicated to the right of each western blot. (**A**) Flag-IP with Flag-tagged USP11 recovers HA-tagged RAE1, (**B**) HA-KCTD6 and (**C**) HA-SPRYD3. (**D**) HA-USP11 co-precipitates with Flag-SPRYD3. (**E**) Flag-tagged RAE1 and KCTD6 co-precipitate HA-tagged USP11. (**F**) Endogenous RAE1, PAM and SPRYD3 co-precipitate with Flag-USP11, whilst no CIT can be detected in the IP western blot. 1% of the extract was loaded in the input; the IP represents 10% of the input. Molecular weight markers are indicated to the left of the western blots. The data shown are representative results from three biological replicates.

To identify novel substrates and/or binding partners of USP11, a U2OS cell line stably expressing Flag-tagged USP11 was generated. Large scale Flag-immunoprecipitation (IP) followed by tandem mass spectrometry (MS/MS) analysis identified peptides of RAE1, potassium channel tetramerization domain containing 6 (KCTD6), Protein associated with Myc (PAM, also known as Myc binding protein 2, MYCBP2), Citron Rho-Interacting Serine/Threonine Kinase (CIT), Ubiquitin Specific Protease 7 (USP7) and SPRY-domain containing protein 3 (SPRYD3), whilst no corresponding peptides were identified in the negative controls that were analysed in parallel (S1 Fig). The majority of the peptides identified in the bands that ran below USP11, belonged to USP11 and are likely degradation products. USP7 and USP11 were previously shown to co-precipitate [7, 27]. Independent reciprocal IPs using Flag- and hemagglutinin (HA)-tagged versions of the proteins transiently expressed in 293T cells, confirmed the interaction between USP11, RAE1, SPRYD3 and KCTD6 (Fig 1A–1E). Antibodies used to IP endogenous proteins were not able to efficiently precipitate the respective endogenous proteins. However, endogenous RAE1, SPRYD3 and PAM co-precipitated with transiently expressed Flag-USP11, whilst CIT binding was not detected (Fig 1F and S7 Fig).

### RAE1 and SPRYD3 are substrates of USP11

**Fig 2:**
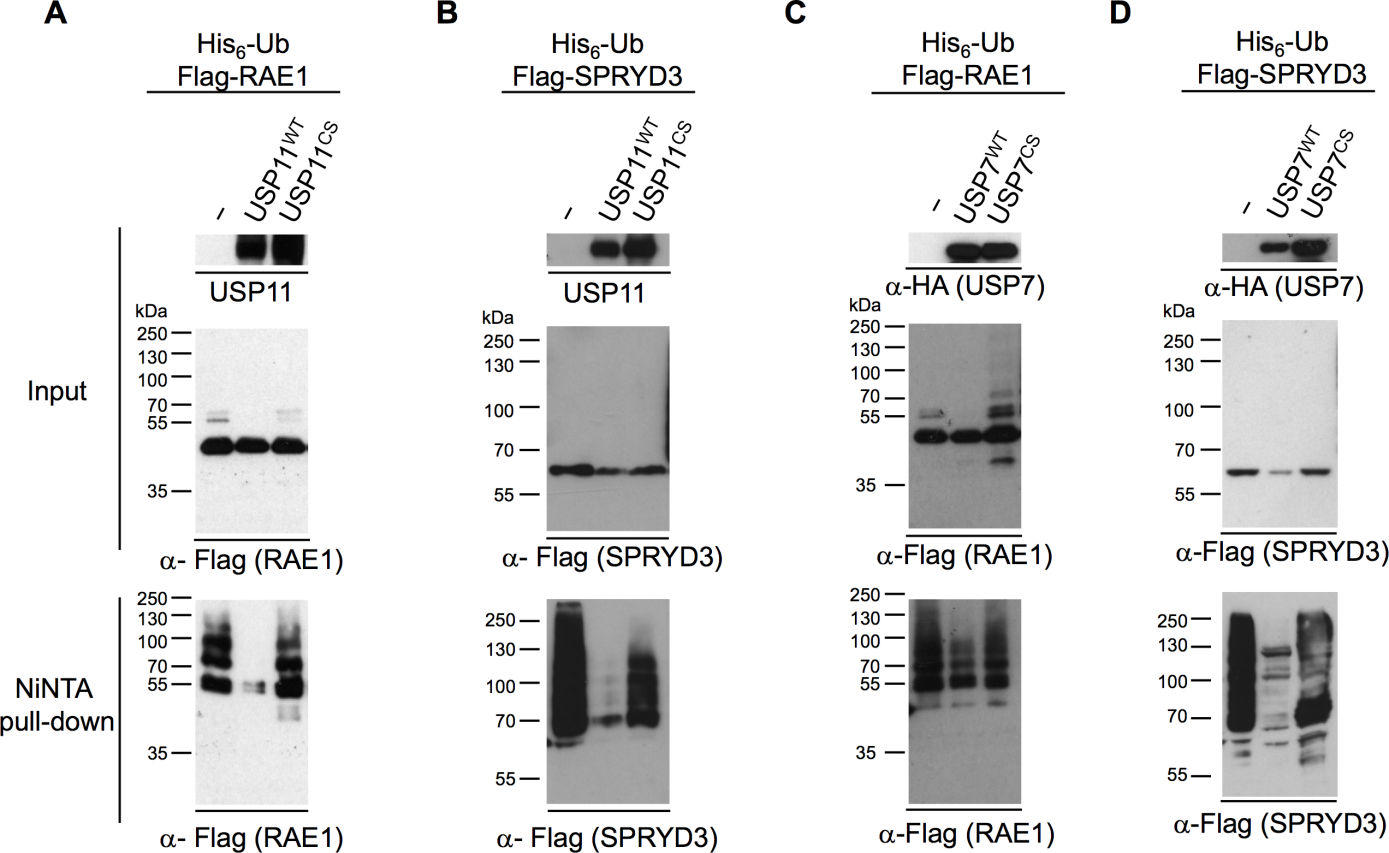
RAE1 and SPRYD3 are substrates for USP11. His_6_-ubiquitin assay; 293T cells were transiently transfected with a His_6_-ubiquitin construct together with Flag-RAE1 (**A** and **C**) or Flag-SPRYD3 (**B** and **D**) and untagged USP11^WT^ or the inactive point mutant, USP11^CS^ (**A** and **B**), HA-tagged USP7^WT^ or HA-USP7^CS^ (**C** and **D**). “–” refers to the empty vector control. Western blots of input and NiNTA pull-down samples are indicated to the left of the panels. The migration of the Mw markers is indicated to the left of the blots. Antibodies used to detect the proteins are indicated under each western blot. These are representative western blots from at least 3 biological replicates.

Since USP11 is a DUB we investigated whether either of these identified binding partners are ubiquitinated and if so whether they are substrates of USP11. Using the His_6_-ubiquitin assay to isolate ubiquitinated proteins from cells [28], we found that ectopically expressed Flag-tagged SPRYD3 and RAE1 are ubiquitinated, whilst KCTD6 is not (S2 Fig). Overexpression of wild type (USP11^WT^) but not catalytic inactive USP11 (USP11^CS^) [7] resulted in deubiquitination of RAE1 and SPRYD3 (Fig 2A and 2B). USP7, which co-IPs with USP11 (S1 Fig, and [7, 27]) and can co-IP USP11, RAE1, PAM and SPRYD3 (S3 Fig), efficiently deubiquitinated SPRYD3, whilst RAE1 is only weakly deubiquitinated by USP7 (Fig 2C and 2D). It is likely that the weak deubiquitination observed for RAE1 upon USP7 overexpression is due to an indirect effect since it associates with USP11 (S1 and S3 Figs and [7]). We were not able to reproducibly detect ubiquitination of the full-length PAM protein. To confirm whether endogenous RAE1 and SPRYD3 are ubiquitinated, we over-expressed His_6_-ubiquitin in 293T cells and concentrated His_6_-ubiquitinated proteins as described above, in the presence or absence of USP11 protein ablation. Unfortunately, the levels of ubiquitinated RAE1 and SPRYD3 in the cell were too low and/or our antibodies lacked the sensitivity to detect endogenous ubiquitinated RAE1 and SPRYD3 in a whole cell lysate.

### Ablation of USP11 or RAE1 reduces cell proliferation of U2OS cells

**Fig 3:**
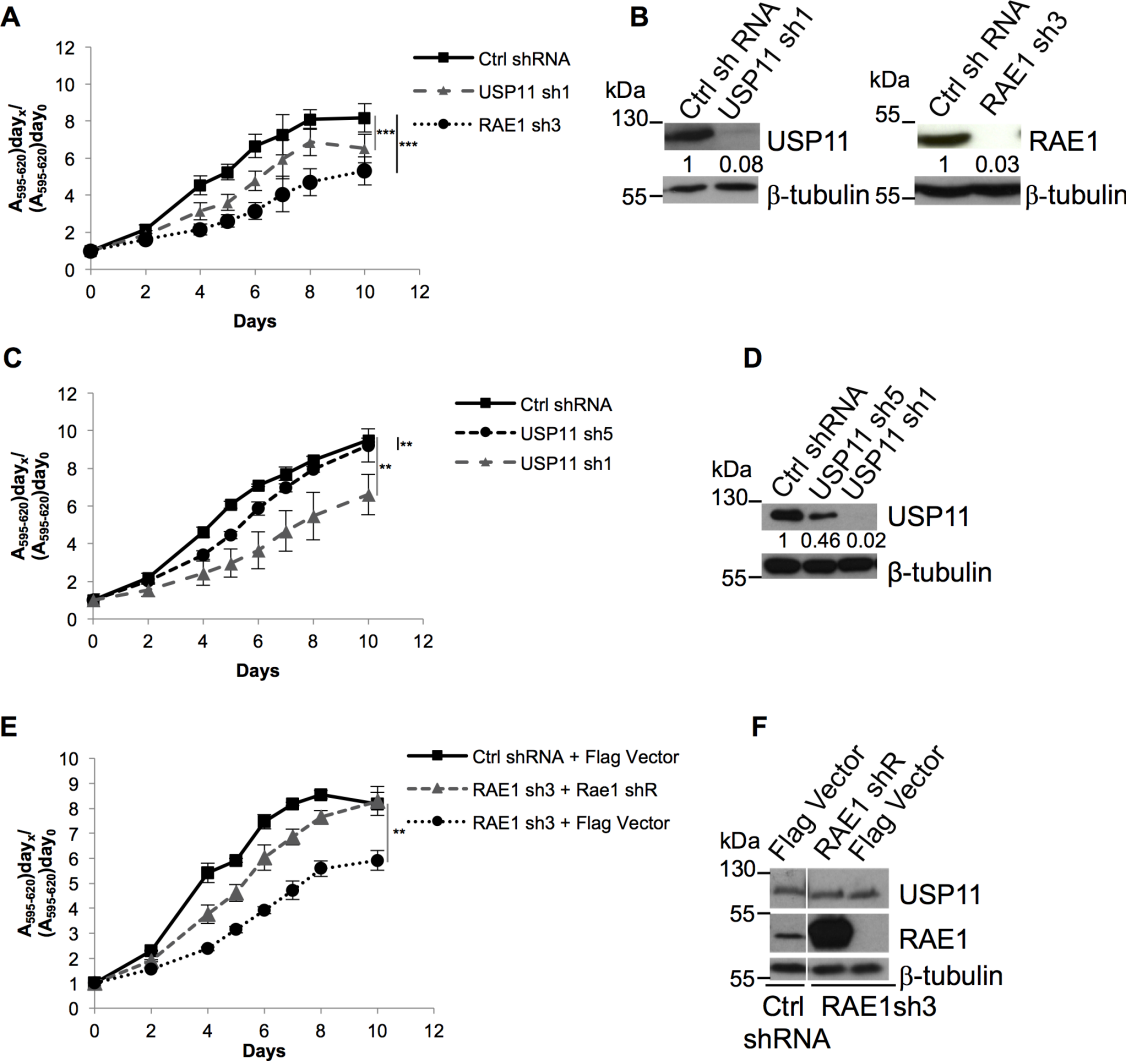
U2OS cell proliferation upon USP11 or RAE1 knock-down. U2OS cell proliferation measured by MTT. (**A**) Knock-down of RAE1 or USP11 significantly reduces cell proliferation compared to control shRNA transduced cells (p = 0.0004; p = 0.0004, respectively). (**C**) MTT cell viability assay measuring cell growth of U2OS cells transduced with control, USP11 sh1 (p = 0.003) or USP11 sh5 (p = 0.0088) shRNA. (**E**) Back-complementation with RAE1 rescues RAE1 shRNA reduced cell proliferation. RAE1shRNA transduced U2OS cells, back-complemented with shRNA resistant RAE1 (RAE1 shR) partially restores growth rates to the control cells (p = 0.0021 for RAE1sh3 + Flag control compared to RAE1sh3 back-complemented with RAE1). (**B**, **D and F**) Western blots illustrating respective protein knock-downs, these are representative for 3 biological replicates. Antibodies used are indicated to the right of each panel. Where blots have been separated by a white line, this indicates that lanes from the western blot, irrelevant to the experiment shown were removed. In panels (**B** and **D**) the numbers shown underneath the USP11 and RAE1 western blots indicate the respective normalized protein levels upon knock-down with the different shRNAs compared to control shRNA treated cells. Molecular weight markers are indicated to the left of each western blot. Averages and SEM of three independent transductions and growth curves are shown. P-values were calculated using the two-tailed paired t-test; compared to the control shRNA transduced cells, and are indicated as follows: **: 0.001 < p < 0.01; ***: p < 0.001.

**Fig 4:**
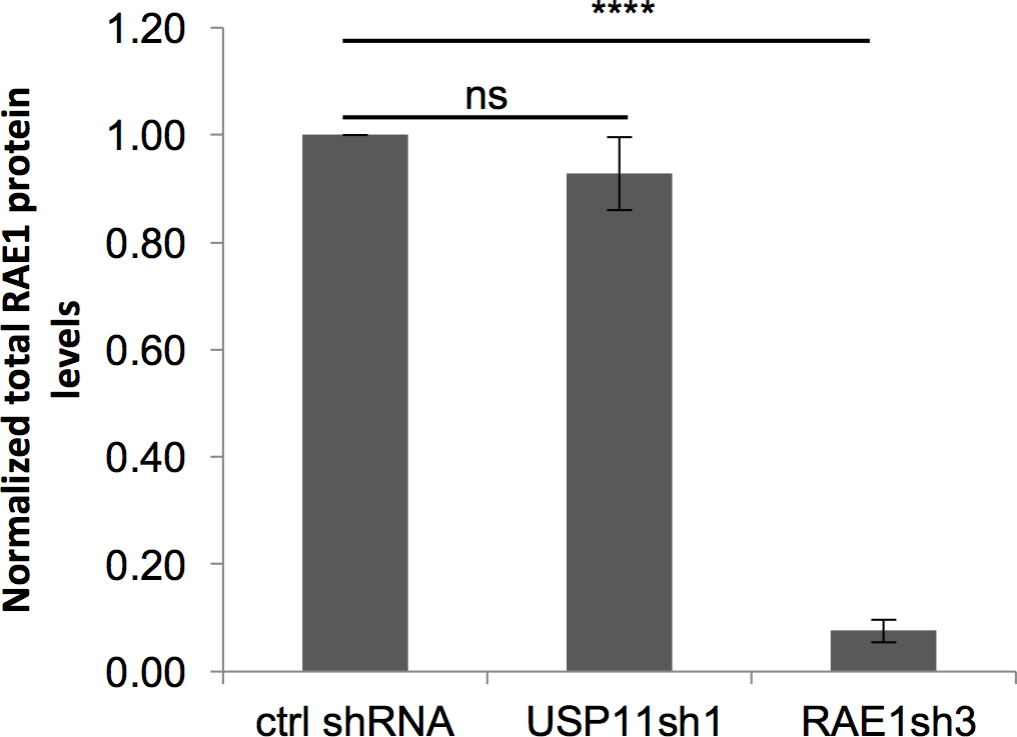
USP11 does not regulate RAE1 protein levels in cells. RAE1 protein levels in the total cell lysate were quantified upon USP11 (n = 5) or RAE1 knock-down (n = 6), normalized to the loading control and compared to the RAE1 protein levels in control shRNA treated cells. Averages and SEM of 5–6 independent transductions are shown. P-values were calculated using the two-tailed paired t-test; compared to the control shRNA transduced cells, and are indicated as follows: ns: not significant, ****: p<0.0001.

We previously showed that knock-down of USP11 in human diploid fibroblasts (HDF) results in p16^INK4a^ mediated growth arrest [7]. Ablation of USP7 also arrested HDFs but in a p16^INK4a^ independent fashion [7]. The INK4A locus in the U2OS cell line is methylated [29], therefore knock-down of USP11 does not result in up-regulation of p16^INK4a^. However, using these previously published lentiviral shRNAs [7], ablation of USP11 in U2OS cells resulted in a reduced cell proliferation measured by both MTT (Fig 3A and 3B) and crystal violet stain (S4 Fig). Importantly, the effect on cell proliferation was dose dependent since the strongest phenotype was observed with the most effective USP11 shRNA (Fig 3C and 3D). Knock-down of RAE1 (Fig 3A and 3B, and S4 Fig) but not SPRYD3 or PAM (S4 Fig), significantly reduced cell proliferation of U2OS cells. Lentiviral shRNA mediated knock-down of USP7 in U2OS cells also reduced cell proliferation, although with USP7sh2 the effect did not reach significance (S4 Fig). Previously, van Deursen and colleagues reported that haplo-insufficient Rae1^+/-^ MEFs did not display any changes in cell proliferation [15]. Thus, to confirm the specific effect observed with RAE1 knock-down, we back-complemented the cells by ectopic expression of RAE1. The lentiviral shRNA targeting the RAE1 transcript (sh3) recognizes the 3'UTR which is absent in our ectopic expression plasmid (S1 Table). Back-complementation partially restored the phenotype confirming the specificity of the RAE1 shRNA knock-down (Fig 3E and 3F). The difference observed between our and previously reported results is likely due to the greater level of knock-down (~ 10 fold, Fig 4 and S5 Fig) we obtain using lentiviral shRNAs compared to the twofold reduction in Rae1 levels in the haplo-insufficient MEFs [15]. We only observed a moderate reduction in cell proliferation, rather than a synergistic effect, when both USP11 and RAE1 expression were ablated compared to the single RAE1 knock-down (S4 Fig), suggesting that USP11 and RAE1 function in the same pathway. Since knock-down of SPRYD3 or PAM in U2OS cells had no significant effect on cell proliferation, we chose to focus on the interaction between USP11 and its substrate RAE1.

Ubiquitination is most known for its role in regulating the life time of proteins in the cell. Indeed, the addition of e.g. Lys48-linked ubiquitin chains targets a protein for proteasomal degradation. To investigate whether USP11 influences the RAE1 protein levels in the cell, we quantified the total RAE1 protein levels upon USP11 knock-down compared to control shRNA treated cells. As a positive control for a reduction in RAE1 protein levels, we transduced the cells with RAE1 shRNA. As can be seen in Fig 4, ablation of USP11 does not significantly change the total RAE1 protein levels in the cell.

### USP11 regulates bipolar spindle formation

Rae1 haplo-insufficiency in mouse embryonic fibroblasts (MEFs) leads to a reduced mitotic index upon treatment with nocodazole, chromosome missegregation and mitotic checkpoint failure [15]. We quantified the mitotic index of nocodazole arrested RAE1, USP11, or control shRNA transduced U2OS cells by MPM2 staining, which detects mitosis specific phosphorylated antigens [30]. As in the Rae1^+/-^ MEFs, knock-down of RAE1 or USP11 in U2OS cells significantly reduced the mitotic index compared to control shRNA treated cells (S6 Fig). Further confirming that ablation of USP11 or RAE1 influences U2OS cell proliferation and not only viability (as measured by the MTT or crystal violet staining in Fig 3 and S4 Fig).

**Fig 5:**
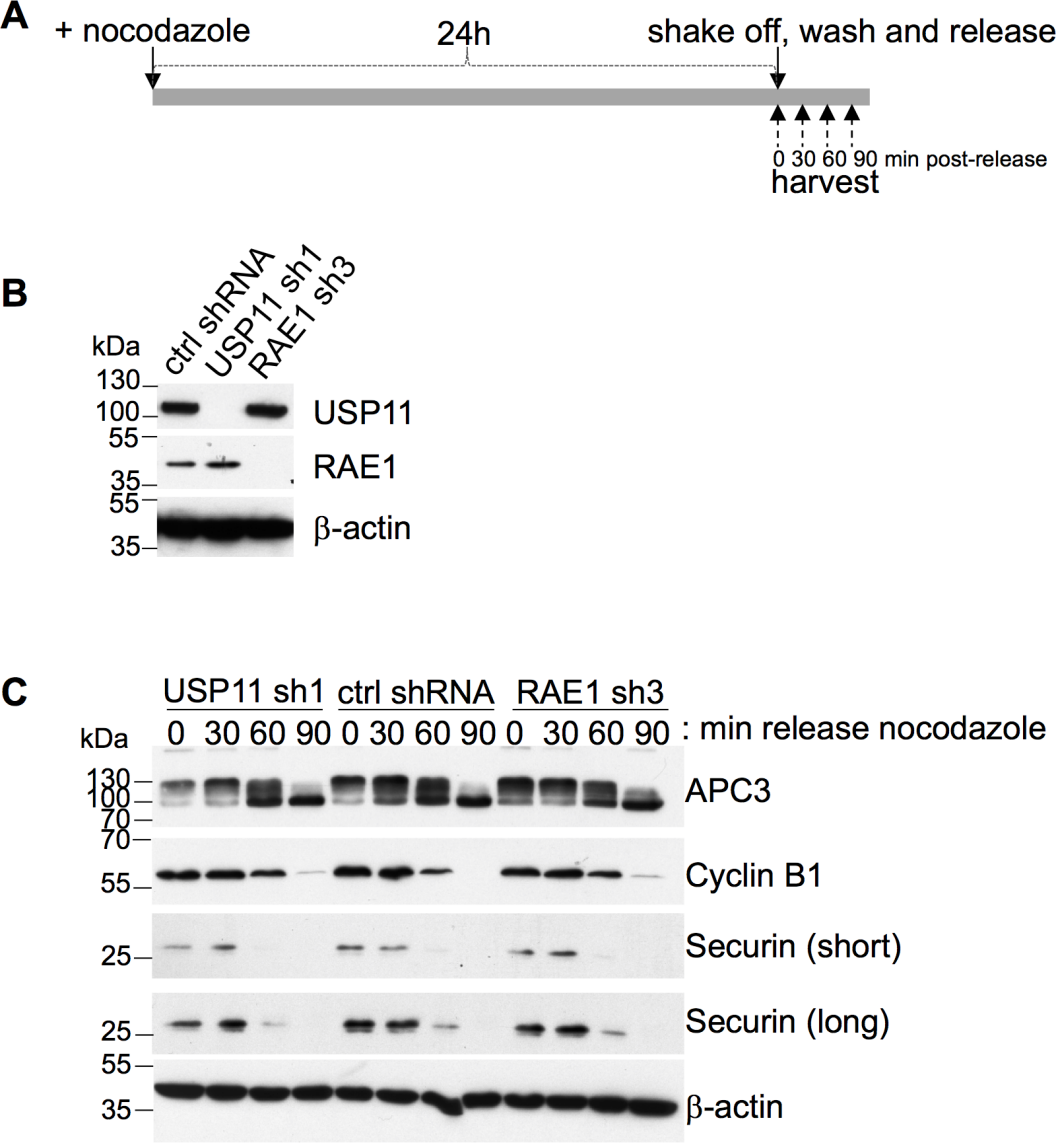
Knock-down of USP11 or RAE1 does not induce premature SAC inactivation. (**A**) Schematic representation of experimental workflow. U2OS cells expressing shRNA targeting USP11 or RAE1 transcripts were incubated for 24h with nocodazole. Mitotic cells were shaken off, washed and released from nocodazole arrest. Samples were taken at indicated time points post-release. (**B**) Western blot verification of USP11 and RAE1 knock-down. (**C**) Western blots illustrating the changes in cyclin B1 and securin protein levels upon knock-down of USP11 or RAE1 compared to control shRNA transduced cells. APC3 dephosphorylation was used as a marker for metaphase to anaphase progression. Antibodies used are indicated to the right of the western blots, molecular weight markers are indicated to the left of the western blot. shRNAs, and time upon release from nocodazole (min) is shown above the western blots. The data shown is representative of 3 biological replicates.

To further investigate whether RAE1 or USP11 play a role in premature SAC inactivation in U2OS cells, we measured the securin and cyclin B1 protein levels at different time points upon release from nocodazole (Fig 5). The progressive dephosphorylation of APC3 was used as a loading control to follow metaphase to anaphase progression (Fig 5) [31, 32]. Both securin and cyclin B1 levels were degraded with similar kinetics in the USP11 and control shRNA treated cells. As was shown previously [18], ablation of RAE1 alone does not result in premature degradation of securin or cyclin B1 (Fig 5).

**Fig 6:**
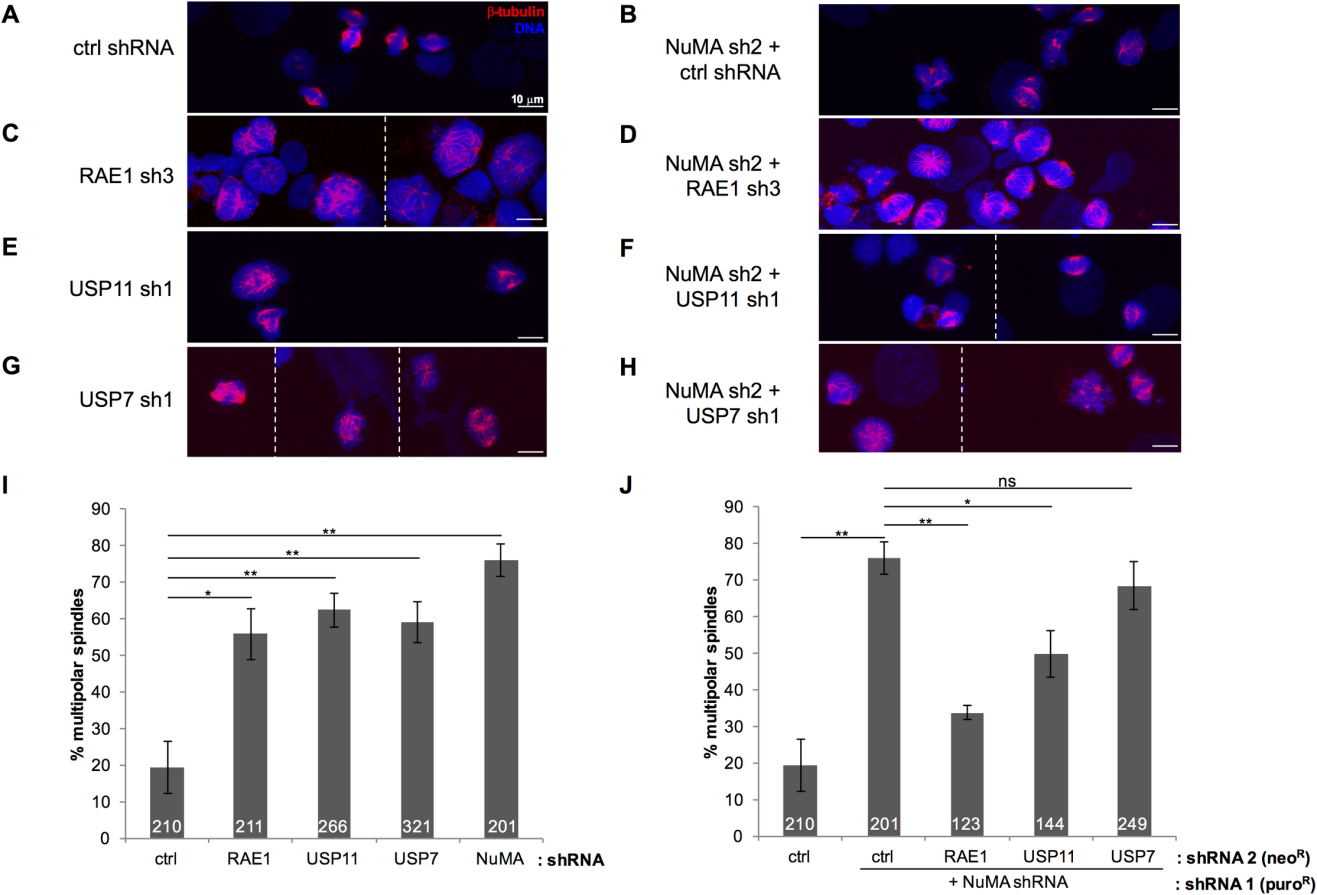
Knock-down of USP11 restores bipolar spindle formation in NuMA shRNA transduced cells. (**A-H**) Representative images of U2OS cells expressing the indicated shRNAs released from nocodazole arrest for 90 min, immunostained for ß-tubulin. Z-stacks were projected into 1 image. The striped line indicates separate images. (**I**) Percentage of multipolar spindles observed in cells expressing the indicated shRNA. The total number of counted cells is indicated in the bars for each shRNA. (**J**) Percentage of multipolar spindles expressing the indicated shRNA in combination with knock-down of NuMA. The total number of counted cells is indicated in the bars for each shRNA. Averages and SEMs are shown; ns: not significant, p > 0.05; * 0.01< p < 0.05; ** 0.001 < p < 0.01. The actual p-values for n biological replicates are, compared to control shRNA treated cells (n = 4): USP11sh1 (n = 4, p-value = 0.0036); USP7sh1 (n = 3, p-value = 0.0070); RAE1sh3 (n = 3, p-value = 0.0155); NuMAsh2 + control shRNA (n = 3, p-value = 0.0014); and compared to NuMAsh2 + ctrl shRNA treated cells: USP7sh1 + NuMAsh2 (n = 2, p-value = 0.457); NuMAsh2 + RAE1sh3 (n = 3, p-value = 0.0043), and NuMAsh2 + USP11sh1 (n = 3, p-value = 0.0317).

We noticed an increase in multi-nucleated cells in which either RAE1 or USP11 expression had been reduced, and wondered whether USP11 plays a role in spindle formation, as was previously shown for RAE1 [22, 25, 26]. Thus, we synchronized the control shRNA treated cells as well as the USP11, RAE1 or NuMA knock-down cells with nocodazole for 24h and released the cells for 90 min into 5 μM MG132, a well-established protocol to arrest cells in metaphase [31–33]. Following fixation and anti-beta-tubulin staining, bi- and multi-polar spindles were quantified (Fig 6). As expected, knock-down of RAE1 (Fig 6C and 6I) or NuMA (Fig 6B and 6I) resulted in a stark increase in the number of cells with defective spindles compared to control shRNA transduced cells (Fig 6A and 6I). Likewise, ablation of USP11 resulted in a similar phenotype (Fig 6E and 6I). As reported before, ablation of both RAE1 and NuMA restored bipolar spindle formation (Fig 6D and 6J). Strikingly, knock-down of USP11 together with NuMA also partially restored the phenotype (Fig 6F and 6J). This suggests that USP11 is involved in regulating the balance between RAE1 and NuMA that can functionally associate at the mitotic spindle. As Giovanizzi *et al*. [34] reported, knock-down of USP7 resulted in a significant increase in multipolar spindles (Fig 6G and 6I). However, unlike USP11, USP7 ablation did not rescue the NuMA shRNA induced phenotype (Fig 6H and 6J).

### USP11 modulates RAE1 ubiquitination at the mitotic spindle

**Fig 7:**
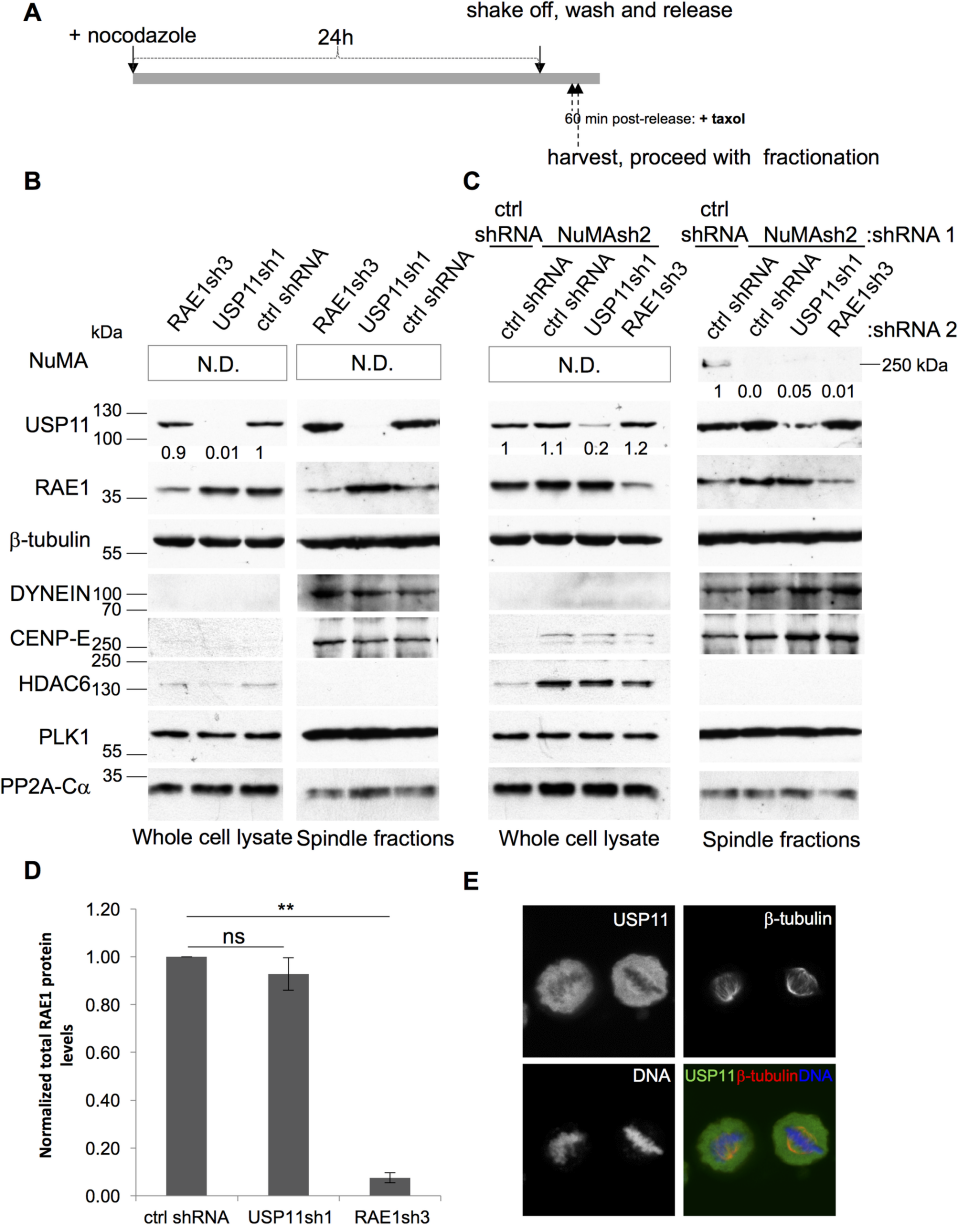
RAE1 and USP11 are associated with the mitotic spindle. (**A**) Schematic representation of experimental workflow of mitotic spindle isolation. U2OS cells expressing indicated shRNAs were treated with nocodazole for 24h. Mitotic cells were washed off, released and treated briefly with taxol before harvesting. Spindle fractionation was done as described previously ([35, 36], see Material and Methods). The western blots shown are representative of the results obtained for three independent biological replicates. (**B**) Purified mitotic spindles were separated by SDS-PAGE (11%) and the indicated proteins were detected by western blot. Knock-down of USP11 does not affect mitotic spindle localization of RAE1. The numbers underneath the USP11 western blot indicate the normalized USP11 protein levels in each sample. (**C**) Purified mitotic spindles from U2OS cells transduced with the indicated shRNAs, were separated on a 7.5% SDS-PAGE gel and transferred onto nitrocellulose for Western blot detection. Representative western blots of protein knock-downs of the transductions used to quantify bi/multi-polar spindles in Fig 6. Antibodies used are indicated to the right of the western blots. Positive controls for mitotic spindle isolation were included (ß-tubulin, DYNEIN, CENP-E, PLK1 and PP2A-Calpha) as well as the chromatin modifier, HDAC6, which was previously shown not to associate with mitotic spindles [35]. Mw markers are indicated to the left of the western blots. The numbers underneath the USP11 and NuMA western blots illustrate the normalized protein levels of USP11 and, respectively, NuMA. (**D**) USP11 does not influence RAE1 association with the mitotic spindle. The amount of RAE1 associated with mitotic spindles upon USP11 or RAE1 knock-down was quantified, normalized to the loading control and compared to cells treated with control shRNA (n = 4). Averages and SEM of four independent knock-down and fractionations are shown. P-values were calculated using the two-tailed paired t-test; compared to the control shRNA transduced cells, and are indicated as follows: ns: p > 0.05; **: 0.0001 < p < 0.001. (**E**) U2OS cells were treated with nocodazole for 24 h, the cells were carefully washed with pre-warmed PBS and treated with 5 μM MG132 for 90 min before fixation. Immunostaining of endogenous USP11, ß-tubulin and DNA was done as described in Materials and Methods.

As USP11 does not regulate the RAE1 protein levels (Fig 4) we wondered whether USP11 modulates the association of RAE1 with the mitotic spindle. Hereto, we isolated mitotic spindles from the shRNA transduced cells used to quantify the bi/multi-polar spindles (Fig 7), using the previously established protocol [35, 36]. As a positive control for mitotic spindle fractionation, we probed our fractions with antibodies that recognize known spindle components [35], such as the structural proteins beta-tubulin, DYNEIN and CENP-E, and the regulatory components PLK1 and PP2A (Fig 7). The chromatin modifying enzyme, HDAC6, which does not co-purify with mitotic spindles [35] was used as a negative control for fractionation. Whilst DYNEIN and CENP-E are only very weakly detected in the whole cell lysate, these structural components are highly enriched in the mitotic spindle fraction (Fig 7). Surprisingly, ablation of USP11 did not change the fraction of RAE1 that co-purifies with mitotic spindles (Fig 7B–7D), and interestingly we can observe that USP11 is also associated with the spindles. Indeed, immunostaining of endogenous USP11 illustrates that a fraction of USP11 is associated with mitotic spindles (Fig 7E). Of note, we observed that ablation of NUMA increases the levels of HDAC6 and CENP-E both in the whole cell lysate and, for CENP-E and DYNEIN, in the mitotic spindle fraction (Fig 7C). Further work is needed to understand how NUMA influences the expression levels and change in mitotic spindle association of these structural proteins.

**Fig 8:**
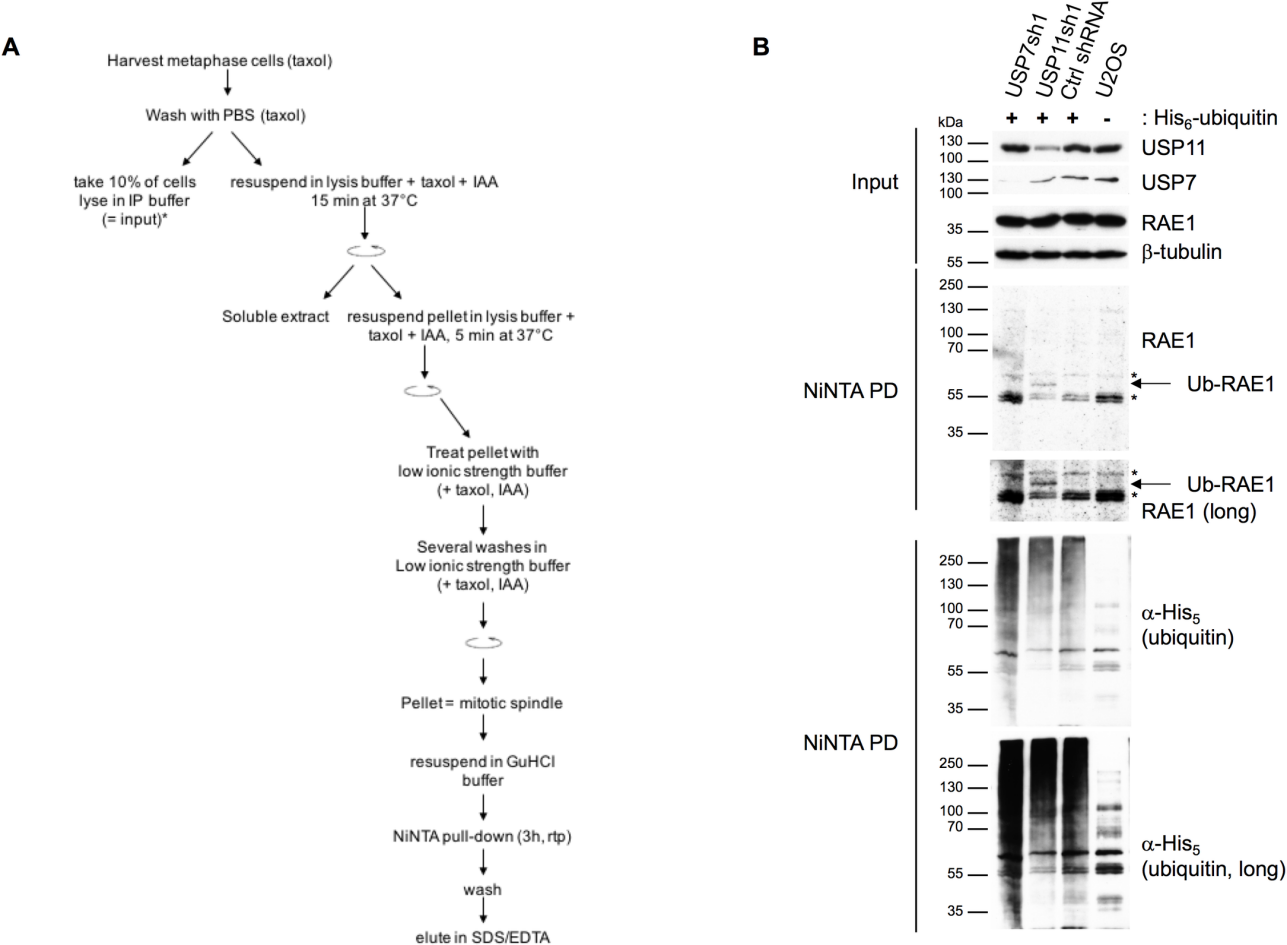
Ablation of USP11 increases ubiquitination of RAE1 on the mitotic spindle. (**A**) Schematic representation of the mitotic spindle fractionation followed by NiNTA pull-down of His_6_-ubiquitinated proteins. (**B**) U2OS cells stably expressing His_6_-ubiquitin were transduced with a control shRNAs or shRNAs targeting USP7 or USP11 transcripts. A U2OS cell line that lacks ectopic expression of His_6_-ubiquitin was used as negative control. After 24h treatment with nocodazole mitotic cells were harvested by shake off, released for 90 min in complete medium supplemented with 5 μM MG132, and treated briefly with taxol (3 min) before harvesting, fractionation was done as shown in (**A**). Samples (input and NiNTA pull-down) were separated on a 7.5% SDS-PAGE gel before transfer onto nitrocellulose membrane. Antibodies used are indicated to the right of each blot; *, indicate non-specific bands.

We speculated that ubiquitination of RAE1, modulated by USP11, could possibly regulate its function at the mitotic spindle. Therefore, we generated a U2OS cell line stably expressing His_6_-ubiquitin. Following mitotic spindle purification, we concentrated the ubiquitinated proteins by NiNTA pull-down under denaturing conditions (Fig 8A). Knock-down of USP11, but not USP7, results in ubiquitination of RAE1 on the mitotic spindle (Fig 8B).

## Discussion

A large-scale mass spectrometry analysis of 75 DUBs, previously detected peptides for USP7, RAE1, PAM and SPRYD3 in Flag-USP11 IPs [27]. Sowa and colleagues indeed confirmed the interaction between USP11 and USP7. We show here that USP11 also binds, directly or indirectly, to SPRYD3, RAE1, PAM and KCTD6.

KCTD6, a BTB/POZ domain (BR-C, ttk and bab/Pox virus and Zinc finger) containing protein, was previously identified as a substrate recruiting sub-domain of a Skp, Cullin, F-box containing (SCF) E3 ligase based on Cullin-3 [37]. Although E3 ligases are often found to be regulated by (auto-)ubiquitination, we could not detect any ubiquitinated forms of KCTD6. Moreover, KCTD6 could also not be co-precipitated with PAM, SPRYD3 or RAE1. Whether KCTD6 and USP11 are functional binding partners is subject of further investigation. SPRYD3 is predicted to have two SPRY domains, typically known to be important for protein-protein interactions [38], and shares 20% homology with RanBPM previously shown to be a substrate of USP11 [39]. Whilst no changes in the endogenous SPRYD3 protein levels can be observed upon USP11 overexpression or knock-down, we could show that ectopically expressed SPRYD3 is extensively ubiquitinated and can be deubiquitinated by USP11. PAM is ubiquitously expressed and is most abundant in cells from the thymus and neuronal lineage [40]. The protein which expresses a C-terminal RING finger domain, was shown to act as an SCF-like E3 ligase with the F-box protein FBXO45, and to be subject to auto-ubiquitination [41, 42]. Interestingly, RAE1 binding to PAM is important for axon termination and synapse formation [43]. We could not detect any changes in RAE1 or USP11 protein levels nor did ablation of PAM or SPRYD3 result in any changes in U2OS cell proliferation (S4 Fig). It will be interesting to see if the PAM: RAE1 (: SPRYD3) complex play additional roles in cell lines different from the neuronal lineage.

Activation of the E3 ligase APC/C^CDH1^ is prevented by interaction of RAE1 and NUP98 with CDH1 during metaphase, thereby preventing ubiquitination of securin and activation of separase [17, 18]. The lack in cell proliferation defect observed by van Deursen and colleagues could be due to the twofold reduction in Rae1 protein levels in Rae1^-/+^ haplo-insufficient MEFs compared to an approximate 10-fold reduction in RAE1 levels achieved with our lentiviral shRNA transduction (Fig 4 and S5 Fig). Our data confirm, however, that a reduction in expression levels of RAE1 alone is not sufficient to change the kinetics of securin degradation (Fig 5, and [18]). As reported previously for RAE1, we also observed a lower mitotic index upon ablation of either USP11 or RAE1 during nocodazole arrest (S6 Fig). Although the effect was less pronounced to previous reports, this could be due to a difference in experimental set-up; Babu *et al*. studied haplo-insufficient Rae1^-/+^ MEFs and quantified the mitotic index by counting H3S10 phosphorylation positive cells [15], whilst we used U2OS cells lentivirally transduced with RAE1 shRNA and quantified the mitotic index by MPM2 staining and FACS.

We show here for the first time that upon USP11 knock-down a ubiquitinated form of RAE1 is present on the mitotic spindle resulting in a phenotype similar to RAE1 ablation, which can be partially restored by combined NuMA and USP11 knock-down. We could only detect a single form of endogenously ubiquitinated RAE1, modified with ~3 ubiquitin molecules derived from its migration on Western blot, and only by using the significantly more sensitive Clarity Max ECL substrate. Additional ubiquitinated forms of RAE1 could possibly occur but might lie yet beneath the sensitivity of the currently available chemiluminescent developing reagents, or could co-migrate with the nonspecific bands detected on the western blots (Fig 8B). Alternatively, the extensive ubiquitination observed in Fig 2 could be due to the overexpression of RAE1.

Given that bipolar spindle formation can be partially restored upon combined USP11 and NuMA knock-down, we hypothesize that ubiquitination of RAE1 interferes with the functional interaction with NuMA at the mitotic spindle. Indeed, ubiquitination is known to influence protein-protein interactions [1]. We also note that the partial rather than the full restoration of combined USP11 and NuMA knock-down could be due to the less efficient knock-down of USP11 in the doubly transduced cells (compare USP11 knock-down efficiency in panels B and C of Fig 7). Recent reports illustrated that an interaction between RAE1 and the cohesin subunit SMC1 is required for proper spindle formation [23, 24]. It will be of interest to investigate whether ablation of USP11 also modulates the functional interaction between SMC1 and RAE1. USP11 is not the first DUB shown to affect mitotic spindle formation. Indeed, confirming previous reports [34, 44], we observed a dramatic increase in multipolar spindles upon ablation of USP7. Moreover, Yan and colleagues reported that the BRCC36 isopeptidase complex (BRICS), like USP11 is associated with microtubules, but influences mitotic spindle assembly by deubiquitinating NuMA [45].

In conclusion, we show here that USP11 is critical for bipolar spindle formation and have identified RAE1 as a novel substrate of USP11. We give evidence that USP11 binds to and regulates ubiquitination of RAE1, hereby likely modulating the functional interaction with the mitotic spindle associated protein NuMA. A model was previously suggested by Wong *et*. *al* [25], where NuMA, through interaction with RAE1 stabilizes and crosslinks microtubule bundles at the negative end of the spindle pole. Here, we suggest that this process is modulated by (de)ubiquitination of RAE1. It will be important to identify the E3 ligase that is responsible for ubiquitinating RAE1 and to determine the spatio- and temporal dynamics of this process. Moreover, it is intriguing that USP7 and USP11 associate with each other in the cell ([7, 27] and this work), influence similar processes ([34] and this work) yet do so by using different substrates.

Bipolar spindle formation and proper segregation of the chromosomes into daughter cells is critical for cellular (and organismal) homeostasis. Whilst aneuploidy is frequently observed in tumours, it remains to be seen whether it is a cause or a result of tumourigenesis. Recent studies suggested that aneuploidy can function as a tumour suppressor or an instigator, depending on the genomic background of the cell [46–49]. It is clear that USP11 ablation can have different effects on cell growth and survival. Previously, we showed that USP11 knock-down in HDFs induces senescence [7], here we report that USP11 ablation in U2OS cells results in multipolar spindle formation with an increased number of multinucleated cells. We attempted to recapitulate observations shown here in cervical cancer cells (HeLa), and noted that all HeLa cells died when transduced with lentiviral USP11 specific shRNAs, days after the puromycin selection had occurred, suggesting a critical role for USP11 in HeLa cancer cell survival. Thus, whilst further investigations are needed to characterize the molecular and structural details of the role both USP11 and RAE1 play in cell proliferation/survival, bipolar spindle formation, chromosome segregation and DNA damage repair (USP11), there could be a niche for USP11 inhibitors as a cancer type specific therapeutic.

## Materials and methods

### Tissue culture

Dulbeccos’s Modified Eagle’s Medium, supplemented with 10% Fetal Bovine Serum (Sigma), 1000 U/ml Penicillin and 1000 μg/ml Streptomycin (Sigma) was used to cultivate Human Embryonic Kidney 293T (referred to as 293T, ATCC) and the U2-osteosarcoma (U2OS, ATCC) cell lines at 37°C, 5% CO_2_ in a humidified atmosphere. Plasmids were transfected in 293T cells by calcium phosphate transfection [50]. Retro-and lentiviral vector production was done as described previously [7]. For lentiviral vector production, the following plasmids were co-transfected: 2 μg pCG-VsVG [51] + 8 μg of pCMV∆8.2 [52] + 10 μg of pLKO-lentiviral shRNA vector (Sigma, S1 Table); for retroviral production: 2 μg pCG-VsVG [51] + 8 μg pCG-GagPol [51] + 10 μg of retroviral plasmid (pQ derived transfer plasmids, S1 Table). U2OS cell lines stably expressing Flag-USP11, or His_6_-ubiquitin were produced by retroviral transduction using the pQFlag-USP11^WT^ puro^R^ or the pQHis_6_-ubiquitin puro^R^ transfer plasmids, cells were selected in the presence of puromycin (0.5 μg/ml (Sigma)). Infections with two lentiviral shRNAs were performed concurrently, and selection was each time started 48h post-transduction (0.5 μg/ml puromycin, 0.5 mg/ml G-418 sulphate (Melford)). All experiments shown reflect biological replicates from independent transductions. Knock-down efficiency was measured every time by western blot. Antibiotic selection was maintained for the duration of the experiment.

### Plasmid construction

Plasmids expressing USP11 and USP7 were previously described [7]. Details on the lentiviral shRNA expression plasmids (Sigma) and plasmid construction of Flag- or HA-tagged RAE1, SPRYD3, KCTD6, and the retroviral His_6_-ubiquitin expression plasmid are summarized in S1 Table. All plasmids were sequence verified.

### Immunoprecipitation

For the large-scale Flag-USP11 IP, U2OS cells stable expressing Flag-USP11 and the parental cell line were grown in 16 x 500cm^2^ dishes. Cells were harvested by trypsinization and washed extensively in ice-cold PBS. Cells were lysed in five volumes of CSK buffer (10 mM Hepes pH 7.4, 10 mM KCl, 1.5 mM MgCl_2_, 0.34 M Sucrose, 100 mM NaCl, 10% glycerol, 1 mM DTT, 1 mM PMSF, 1 x complete EDTA free protease inhibitor (Sigma)) supplemented with 0.5% NP40 (Sigma) for 10 min on ice. After centrifugation, the supernatant was pre-cleared over Protein A agarose beads (GE Healthcare) and then incubated with anti-Flag agarose beads (Sigma) by end-over-end rocking at 4°C for 4h. After extensive washes, proteins were eluted with CSK buffer supplemented with 0.1 mg/ml Flag peptide (Sigma) for 30 min. The eluate was precipitated with trichloroacetic acid before separation by SDS-PAGE. Bands were excised in the Flag-USP11 lane and at exactly the same position for the negative control lane which ran in parallel, and sent for MS/MS analysis. We used the following filters to determine which proteins specifically co-purified with Flag-USP11: (i) proteins of which peptides were present in the negative control sample were discarded; (ii) the datasets were compared with previous MS/MS analysis of Flag-tag purifications performed on unrelated proteins (7 independent data sets used including [7, 53–55]) and the CRAPOME database [56], proteins that typically co-purify using the used anti-Flag resin were removed from the list of potential binding partners; (iii) identified proteins were considered positive if their molecular weight compared to the estimated molecular weight based on the migration of the band that was excised from the gel, and (iv) of the remaining proteins we only considered those significant if more than 3 true tryptic peptides were identified in at least one of the independent experiments. Small scale IPs were done in 293T cells. Hereto, the indicated plasmids were transfected into 293T cells using the calcium phosphate precipitation method [50]. Where two or more plasmids were co-transfected, equal amounts of each plasmid were used. For negative control experiments, the parental plasmid without insert was used. Transfected cells were harvested 36h post-transfection and the IP was done as described above.

### Ubiquitination assay and mitotic spindle isolation

Enrichment of ubiquitinated proteins [7, 28] and isolation of mitotic spindles were done as previously described [36]. Four 15 cm dishes of lentiviral shRNA transduced U2OS cells were used for each spindle purification. Briefly, cells were synchronized in M phase by treatment with 200 ng/ml nocodazole for 24h. Cells were harvested by mitotic shake off, washed extensively in room temperature PBS and resuspended in pre-warmed complete medium and put back in the 37°C, 5% CO_2_ humidified incubator. Progression to metaphase was monitored by taking small aliquots and staining the cells with the following staining solution: 3% formaldehyde, 2% sucrose, 0.2% Triton X-100, 4 μg/ml 4',6-diamidino-2-phenylindole (DAPI). Once the large majority of the cells reached metaphase, 5 μg/ml taxol was added and the cells were harvested 3 min later. All procedures were done at room temperature unless otherwise stated. Cells were centrifuged at 300 *g*, washed with 10 ml PBS supplemented with 1 mM PMSF, 5 μg/ml taxol. Cells were then lysed in 0.5 ml lysis buffer (100 mM Pipes pH 6.9, 1 mM MgSO_4_, 2 mM EGTA, 0.5% NP-40, 5 μg/ml taxol, 200 μg/ml DNAse I (Sigma), 10 μg/ml RNAseA (Sigma), 1 U/ml micrococcal nuclease (New England Biolabs), 20 U/ml benzonase (Sigma), Complete EDTA free protease inhibitor (Roche), and 20 mM ß-glycerophosphate). The resuspended cells were incubated at 37°C for 15 min and stirred every 2 min by swirling the tube. The lysis was cleared by centrifugation at 700 *g* for 2 min. The pellet was resuspended in the same lysis buffer and incubated for a further 5 min at 37°C. The sample was centrifuged and the supernatant removed. The walls of the tube were washed with 0.5 ml low-ionic strength buffer (1 mM Pipes pH 6.9, 5 μg/ml taxol, Complete EDTA free (Roche) without disturbing the pellet. The pellet was then resuspended in 0.5 ml of this low-ionic strength buffer and incubated at room temperature for 10 min. The spindles were harvested by centrifugation (3 min 1500 *g*). The supernatant was removed and the pellet was resuspended again in low ionic strength buffer, and the pellets were collected by centrifugation at 1500 *g*. Purified spindles were boiled in 1% SDS, protein concentration was determined by Bradford assay and the samples were separated on 11% or 7.5% (when also blotting for NuMA) SDS-PAGE gels. For the isolation of ubiquitinated proteins that co-purify with the spindle, the U2OS cell line that stably expresses His_6_-ubiquitin was transduced with lentiviral shRNAs targeting the USP7, USP11 or no mammalian transcripts (control shRNA) [7]. Following synchronization in M phase, mitotic cells were harvested by shake off, washed three times in room temperature PBS and resuspended in pre-warmed (37°C) complete medium supplemented with 5 μM MG132. Isolation of mitotic spindles was carried out as described above with the single modification that 10 mM iodoacetic acid (IAA) was added to each buffer to inhibit Cys-proteases. Purified spindles were resuspended in denaturing buffer (6 M Guanidine-HCl, 0.1 M Na_2_HPO_4_/NaH_2_PO_4,_ pH 8.0, 10 mM imidazole with NaOH) and His_6_-ubiquitinated proteins were purified by NiNTA agarose as described above. A fraction of the harvested cells was lysed in IP buffer to normalize the input in the NiNTA pull-down.

### Growth curves

Proliferation of U2OS cells with depletion of specific proteins was monitored using 3-[4,5-dimethylthiazol-2-yl]-2,5-diphenyl tetrazolium bromide (MTT, Sigma) [57] and crystal violet staining [58]. For each experiment, independent lentiviral shRNA transductions were carried out. Puromycin (0.5 μg/ml, Sigma) was added 2 days post-infection. Two days later, 1 000 cells/well were plated in a 96-well plate, antibiotic selection was maintained. For each time point 12 wells were plated. Measurements were normalized to day 0 (= 24h post-plating out the cells in 96 well plates). Western blot analysis verifying the knock-downs were done using extracts made on day 0 (shown) and day 8 of the growth curves. Growth curves show the averages and standard error of mean (SEM) from 3 biological replicates. P-values were calculated using the two-tailed paired t-test.

### Flow cytometry analysis

U2OS cells transduced with lentiviral shRNAs were treated with 100 ng/ml nocodazole for the indicated time points. Cells were harvested by trypsinization, washed with PBS and fixed in 70% ethanol. To determine the mitotic index, we stained and processed the cells with the anti-MPM2 antibody (Millipore) as described previously [30]. The samples were analysed by FACS. Negative control samples (unstained synchronized cells and stained cycling cells) were used to set the gates. Single, live cells were gated and analysed for MPM2 positive signal. 10 000 events were counted. Averages and standard error of mean (SEM) are shown for three biological replicates. P-values were calculated using the student t-test.

### Immunostaining and confocal imaging

U2OS cells transduced with lentiviral shRNAs were plated out in 2-well nunc slides to reach ~50% confluence the next day when they were treated with 200 ng/ml nocodazole for 24h. The cells were released by extensive, but careful, washing with PBS not to lose the mitotic cells from the slides, and the medium was replaced containing 5 μM MG132 for 90 min. Cells were then fixed and immunostaining was done as described previously [53] with the difference that all incubations were done at room temperature. The following antibodies were used: rabbit anti-USP11 ([7], 1:300), mouse anti-beta tubulin (Sigma, 1:500), secondary goat anti-mouse Alexa-555 conjugated antibody (Molecular Probes, 1:500), goat anti-rabbit Alexa-488 conjugated antibody (Cell Signaling Technology, 1:500). DNA was visualized using DRAQ5^TM^ (Biolegend) at 2.5 μM final concentration. Z-stacks (0.42 μm slices) were taken on a LSM510 confocal microscope (Zeiss) using a Plan-Apochromat 63x/1.4 Oil DIC objective with scan zoom of 1. Eight averages were taken of every image and the resolution was set to 1024x1024. Alexa-555 was detected through a 560–615 nm BP filter, DRAQ5^TM^ was detected through a LP 650 nm filter. Z-stacks were projected into 1 image. P-values were calculated using the paired student t-test.

### Western blot and antibodies

Western blot detection was done as described previously [7]. The following antibodies were used: rabbit anti-RAE1 (Sigma, 1:2000), mouse anti-beta tubulin (Sigma, 1:2000), mouse anti-Flag horse radish peroxidase (HRP) conjugated antibody (Sigma, 1:5000), rabbit anti-SPRYD3 (Abcam, 1:2000), rabbit anti-CIT (CT295, 1:1000)[59] (kindly provided by Prof Kennedy of the California Institute of Technology), rabbit anti-PAM (Bethyl Laboratories A301-833A, 1:2000), rabbit anti-KCTD6 (Sigma), rabbit anti-USP11 [7] (1:2000), mouse anti-beta actin HRP (Abcam, 1:20000), mouse anti-hemagglutinin (HA) clone 16B12 (Covance, 1:2000), rabbit anti-HA (Sigma, 1:1000), mouse anti-securin (Novus Biologicals, 1:500), mouse anti-cyclin B1 (Santa Cruz Biotechnology, 1:2000), rabbit anti-APC3 (Cell Signalling Technologies, 1:1000), rabbit anti-NuMA (Cell Signalling Technologies, 1:1000), mouse anti-HDAC6 (Insight Biotechnology Ltd, 1:200), mouse anti-DYNEIN (Santa Cruz Biotechnology, 1:100), mouse anti-CENP-E (Insight Biotechnology Ltd, 1:200), rabbit anti-Plk1 (Bethyl Laboratories). Donkey anti-rabbit and sheep anti-mouse HRP conjugated antibodies (GE Healthcare) were used at a 1:2000 dilution. For the detection of NuMA and the ubiquitination of endogenous RAE1, Clarity Max ECL substrate was used, all other western blots were detected with Clarity ECL substrate (Bio-Rad).

## Supporting information

S1 Table Plasmids and primer sequences used in this manuscript. (**A**) Lentiviral shRNA constructs. (**B**) Eukaryotic expression plasmids. (**C**) Primer sequences.
(PDF)

S1 Fig Tandem mass spectrometry results. **(A, B)** Colloidal Coomassie stained gels of Flag-USP11 purification. Migration of USP11 is indicated on the gels. The migration of the Mw standards is indicated to the left of the gels. **(A)** Bands A and B, together with C and D (negative control, Flag-IP performed on extracts of the parental 293T cell line run in parallel) were analyzed by tandem MS. **(B)** Bands A-M (Flag-USP11) and N-Z (negative control) were analyzed by tandem MS. Most bands identified in the C-M bands belonged to USP11. **(C)** Table with results of tandem MS analysis. Only proteins for which no peptides were identified in the negative control lane are shown.
(TIF)

S2 Fig Ubiquitination of SPRYD3, RAE1, and KCTD6. NiNTA pull-down of His_6_-ubiquitinated proteins under denaturing conditions [28]. 293T cells were transfected with pMT107 and (**A**) Flag-SPRYD3, (**B**) HA-KCTD6 or HA-RAE1. Antibodies used are indicated underneath each western blot.
(TIFF)

S3 Fig Flag-IP of Flag-USP7 transiently expressed in 293T cells. Detection of endogenous proteins are indicated to the right of each western blot. Input and IP are indicated above the western blots. The negative control sample comes from 293T cells transfected with the empty pQFlag-puroR plasmid. 1%, respectively, 10% of the input and IP samples were separated on gel.
(TIFF)

S4 Fig Knock-down of SPRYD3 or PAM in U2OS cells does not alter cell proliferation. (**A**) Growth curve of U2OS cells transduced with control, USP11sh1 or RAE1sh3 RNA measured by crystal violet staining. The knock-down of RAE1 or USP11 results in a significant growth defect compared to the control shRNA transduced cells (p = 0.0167; p = 0.042, respectively). (**C**) Ablation of PAM or SPRYD3 does not significantly change the proliferation of U2OS cells as measured by MTT assay (p = 0.8575; p = 0.05, respectively). (**E**) Cell viability measured by MTT assay. Knock-down of USP7 reduces cell proliferation compared to control shRNA transduced cells (p = 0.0285 for USP7sh1; p = 0.0547 for USP7 sh2). (**G**) Cell proliferation measured for U2OS cells transduced with control, USP11sh1, RAE1sh3 or USP11sh1+RAE1sh3 shRNAs as indicated in the legend. No synergistic growth effect was observed by simultaneous ablation of USP11 and RAE1. The cells did, however, grow significantly slower than cells with ablation of RAE1 only (p = 0.0019). Averages and SEM of three independent transductions and growth curves are shown. p-values were calculated using the two-tailed paired t-test, compared to the control shRNA transduced cells, and are indicated as follows: ns: p > 0.05; *: 0.01< p < 0.05; **: 0.001 < p < 0.01; ***: p < 0.001. (**B**, **D**, **F, H**) Western blots illustrating respective protein knock-downs. For each of the panels shown, samples were analyzed on the same western blot. Where a white line is shown, this is to indicate that some lanes, irrelevant to the experiment shown, were removed from the Figure. Antibodies used are indicated to the right of each panel.
(TIF)

S5 Fig Measuring knock-down efficiency of RAE1 and USP11. Semi-quantitative western blot analysis of RAE1 and USP11 protein levels illustrates that RAE1sh3 reduces RAE1 protein levels in U2OS cells ~ 10 fold, whilst USP11 protein levels are reduced more than 10 fold. Titration of control shRNA transduced cells as indicated above the western blot. Antibodies used are shown at the right of the western blots. 50 μg of total protein extract was loaded for the USP11 sh1 and RAE1 sh3 transduced cells.
(TIFF)

S6 Fig USP11 or RAE1 knock-down reduces the mitotic index of U2OS cells. (**A**) U2OS cells transduced with the indicated shRNAs were arrested with 100 ng/ml nocodazole (or DMSO as negative control “0h”). Cells were harvested 18h or 24h post-treatment and fixed with 70% ethanol, or extensively washed in PBS and released into preheated complete medium following 24h nocodazole treatment (“4h release”). The mitotic index was determined by FACS analysis using MPM2 staining as an indicator of mitotic cells. Less mitotic U2OS cells were measured upon knock-down of USP11 or RAE1 after 18h (p = 0.0126; p = 0.0022, respectively) and 24h nocodazole treatment (p = 0.008; p = 0.0063, for USP11 and RAE1, respectively) in comparison to the control shRNA transduced cells. (**B**) Western blot analysis of protein levels during nocodazole arrest and after 4h recovery (grey line). All samples were analyzed on the same blot, and are separated here by a white line for clarity. Antibodies used are indicated to the right of the western blots.
(TIFF)

S7 Fig Scans of whole western blots of Fig 1F. Flag-USP11 IP. Samples were loaded twice and probed with different antibodies. Upper 3 images are different exposure times of the same western blot; bottom 2 are 2 different exposure times of the same western blot. The grey/white striped lines indicate where blots were cut before antibody probing. Antibodies used are indicated to the right of the blots, molecular weight markers to the left. Exposure times are indicated above the western blot. The boxed areas on the western blots indicate which exposures were shown in Fig 1F.
(TIFF)
