# Supplementary material for: USP11 deubiquitinates RAE1 and plays a key role in bipolar spindle formation
Source: PLoS One. 2018 Jan 2;13(1):e0190513. doi: 10.1371/journal.pone.0190513 (PMC5749825; doi:10.1371/journal.pone.0190513)
Supplement: S1 Table — (A) Lentiviral shRNA constructs. (B) Eukaryotic expression plasmids. (C) Primer sequences. (PDF) [file pone.0190513.s001.pdf]

Supplementary Table S1 : constructs and primers used in this manuscript

a. Lentiviral shRNAs

Name	Based on	Target	source/reference
**pLKO- SPRYD3 shRNA2 puroR**	NM_032840.1-710s21c1	CDS	Sigma
**pLKO-USP11 shRNA1 puroR**	NM_0046512 1800	CDS	Sigma
**pLKO-USP11 shRNA5 puroR**	Created with GNM151 + GNM152	3'UTR	
**pLKO-RAE1 shRNA3 puroR**	NM_003610.3-1572s1c1	3'UTR	Sigma
**pLKO-PAM shRNA1 puroR**	NM_015057.3-14495s21c1	3'UTR	Sigma
**pLKO-USP7 shRNA1 puroR**	NM_003470 1249	CDS	Sigma/[1]
**pLKO-USP7 shRNA2 puroR**	NM_003470 1648	CDS	Sigma/[1]
**pLKO-NuMA shRNA2 puroR**	NM_006185.2-5966s21c1	CDS	Sigma
**pLKO- ctrl shRNA puroR**	SHC002	no mammalian target	Sigma/[1]
**pLKO-ctrl shRNA neoR**	SHC002 puroR cassette replaced by neoR	no mammalian target	primers : GNM244/245
**pLKO-USP11 shRNA1 neoR**	pLKO-USP11 shRNA1 puroR - puroR cassette replaced by neoR	CDS	primers : GNM244/245
**pLKO-RAE1 shRNA3 neoR**	pLKO-RAE1 shRNA3 puroR - puroR cassette replaced by neoR	3'UTR	primers : GNM244/245

b. Eukaryotic expression plasmids

Name[$]	Description (expression of)	primers	template	destination plasmid
**pQHA-RAE1 puroR**	HA-tagged RAE1	GNM81 + GNM82	HeLa cDNA	pQHA-puroR[1]
**pQFlag-RAE1 puroR**	Flag-tagged RAE1	AgeI/BamHI insert from pQHA-RAE1	pQHA-RAE1 (this study)	pQHA-RAE1 (this study)
**pQHA-KCTD6 puroR**	HA-tagged KCTD6	GNM84 + GNM85	HeLa cDNA	pQHA-puroR[1]
**pQFlag-KCTD6 puroR**	Flag-tagged KCTD6	AgeI/BamHI insert from pQHA-KCTD6	pQHA-KCTD6 (this study)	pQHA-KCTD6 (this study)
**pQFlag-USP11sh1R-hygroR**	Flag-USP11 shRNA sh1 resistant	GNM246+GNM247	pQFlag-USP11 puroR	pQFlag-hygroR[2]
**pQFlag-RAE1-hygroR**	Flag-RAE1 shRNA sh3 resistant	AgeI/BamHI insert from pQFlag-RAE1		pQFlag-hygroR[2]
**pQFlag-BUB3 puroR**	Flag-tagged BUB3	GNM280+GNM281	HeLa cDNA	pQFlag-puroR[1]
**pQFlag-USP11^{WT} puroR**	Flag-USP11^{WT}			1
**pQHA-USP11^{WT} puroR**	HA-USP11^{WT}			1
**pQFlag-USP11^{WT} hygroR**	Flag-USP11^{WT}	GNM243 and GNM26	pQFlag-USP11 puroR	pQFlag-hygroR[2]
**pQ-USP11^{WT} puroR**	untagged USP11^{WT}	GNM13 + GNM26	pQFlag-USP11^{WT} puroR	
**pQ-USP11^{CS} puroR**	untagged USP11^{CS} (C275S)		pQFlag-USP11^{CS} puroR	
**pQHis$_6$-Ubiquitin puroR**	His$_6$-ubiquitin	NotI/EcoRI digest from pMT107[3]		pQCXIP (Clontech)
**pMT107**	His$_6$-ubiquitin			3
**pGMFlag-SPRYD3**	Flag-tagged SPRYD3	GNM86 + GNM87	HeLa cDNA	pGM-Flag[4]
**pCG-GagPol**	retroviral packaging plasmid			5
**pCG-VsV-G**	Vesicular stomatitis Virus G			5
**pCMV-Δ8.2**	lentiviral packaging plasmid			6

[$] all pQ named plasmids are based on pQCXIP (Clontech) and are retroviral expression plasmids

References

1 Maertens, G.N., S. El Messaoudi-Aubert, S. Elderkin, K. Hiom, and G. Peters. 2010. Ubiquitin-specific proteases 7 and 11 modulate Polycomb regulation of the INK4a tumour suppressor. EMBO J. 29:2553-2565.

2 Maertens, G.N., N.J. Cook, W. Wang, S. Hare, S.S. Gupta, I. Oztop, K. Lee, V.E. Pye, O. Cosnefroy, A.P. Snijders, V.N. KewalRamani, A. Fassati, A. Engelman, and P. Cherepanov. 2014. Structural basis for nuclear import of splicing factors by human Transportin 3. PNAS. 111:2728-2733.

3 Treier, M., L.M. Staszewski, and D. Bohmann. 1994. Ubiquitin-dependent c-Jun degradation in vivo is mediated by the delta domain. Cell. 78:787-798.

4 Maertens, G.N., P. Cherepanov, and A. Engelman. 2006. Transcriptional co-activator p75 binds and tethers the Myc-interacting protein JPO2 to chromatin. J Cell Sci. 119:2563-2571.

5 Ulm, J.W., M. Perron, J. Sodroski, and C.M. R. 2007. Complex determinants within the Moloney murine leukemia virus capsid modulate susceptibility of the virus to Fv1 and Ref1-mediated restriction. Virology. 363:245-255.

6 Naldini, L., U. Blomer, F.H. Gage, D. Trono, and I.M. Verma. 1996. Efficient transfer, integration, and sustained long-term expression of the transgene in adult rat brains injected with a lentiviral vector. Proc Natl Acad Sci U S A. 93:11382-11388.

c. Primer sequences

Primer	Sequence 5'-3'	Description
**GNM81**	GGCCACCGGTATGAGCCTGTTTGGAAC	sense - RAE1 - with AgeI site
**GNM82**	CCGGCTCGAG CTA CTTCTTATTCCTGGGC	antisense - RAE1 (with stop codon)- BamHI site
**GNM84**	CCGGACCGGTATGGATAATGGAGACTGGG	sense - KCTD6 - AgeI site
**GNM85**	GGCCGGATCCTCAGTCGTCTGTCTTCCG	antisense - KCTD6 (with stop codon) - BamHI site
**GNM86**	GGCCTGATCAATGAGGAGGACGCGG	sense - SPRYD3 - BclI site
**GNM87**	GGCCGAATTCCTAGCCACTCAAGGGG	antisense - SPRYD3 (with stop codon) - EcoRI site
**GNM151**	CCGGTCTGAGTTCATGGATGTTAATTCTCGAGAATTAACATCCATGAACTCAGTTTTTTG	sense - USP11 shRNA 5
**GNM152**	AATTCAAAAAACTGAGTTCATGGATGTTAATTCTCGAGAATTAACATCCATGAACTCAGA	antisense - USP11 shRNA 5
**GNM243**	GGCCACCGGTATGGCAGTAGCCCCGCGACTG	sense - USP11 (start aa1) - AgeI site
**GNM246**	CCTGCGGGAGCGCACCCCTGCACGGGATTATAATAATTCATACTACGGCCTGATGCTTTTT	sense for mutagenesis -USP11 shRNAsh1 resistant
**GNM247**	CCAAAAAGCATCAGGCCGTAGTATGAATTATTATAATCCCGTGCAGGGGTGCGCTCCCGC	antisense for mutagenesis -USP11 shRNAsh1 res.
**GNM280**	GGCCCTCGAG ATGACCGGTTCTAACGAGTTCAAGC	sense - BUB3 - XhoI site
**GNM281**	GGCCGAATTCTCAAGTACATGGTGACTTGGGTTTTG	antisense -BUB3 (with stop codon - EcoRI site
**GNM13**	GGCC GAATTC ATGGCAGTAGCCCCGCG	sense - untagged USP11 - EcoRI site
**GNM26**	CCGCTCGAGTCAATTAACATCCATGAACTCAGAGC	antisense - USP11 with Stop codon - XhoI site
**GNM244**	CCGG GGATCC ATGATTGAACAAGATGGATTGCACG	sense nemocyin resistance cassette - BamHI site
**GNM245**	GGCC GGTACC TCAGAAGAACTCGTCAAGAAGGCG	antisense neomycin resistance cassette - stop - KpnI
