# Supplementary figures and images for: USP11 deubiquitinates RAE1 and plays a key role in bipolar spindle formation
Source: PLoS One. 2018 Jan 2;13(1):e0190513. doi: 10.1371/journal.pone.0190513 (PMC5749825; doi:10.1371/journal.pone.0190513)

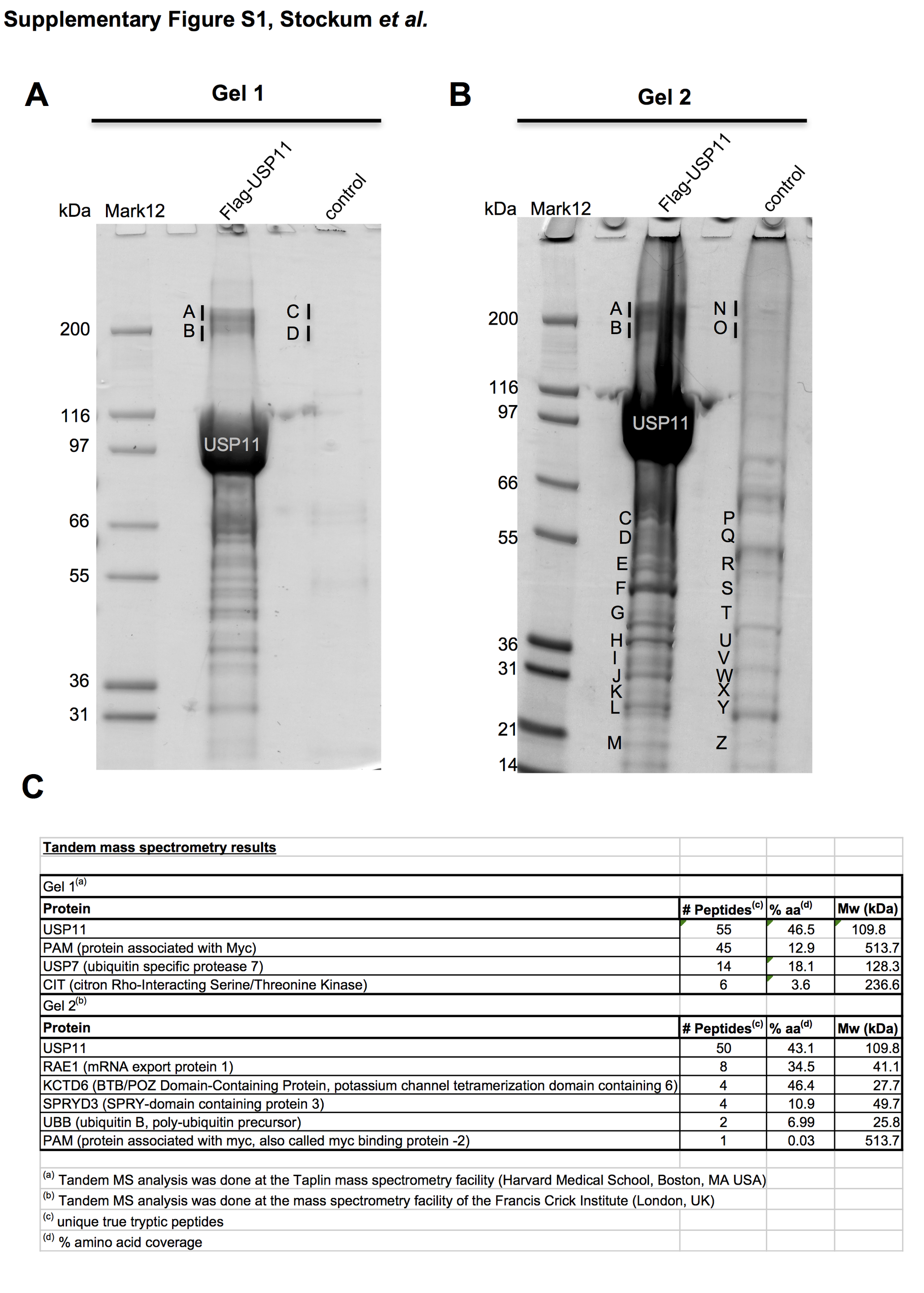

Supplement: S1 Fig — (A, B) Colloidal Coomassie stained gels of Flag-USP11 purification. Migration of USP11 is indicated on the gels. The migration of the Mw standards is indicated to the left of the gels. (A) Bands A and B, together with C and D (negative control, Flag-IP performed on extracts of the parental 293T cell line run in parallel) were analyzed by tandem MS. (B) Bands A-M (Flag-USP11) and N-Z (negative control) were analyzed by tandem MS. Most bands identified in the C-M bands belonged to USP11. (C) Table with results of tandem MS analysis. Only proteins for which no peptides were identified in the negative control lane are shown. (TIF) [file pone.0190513.s002.tif]

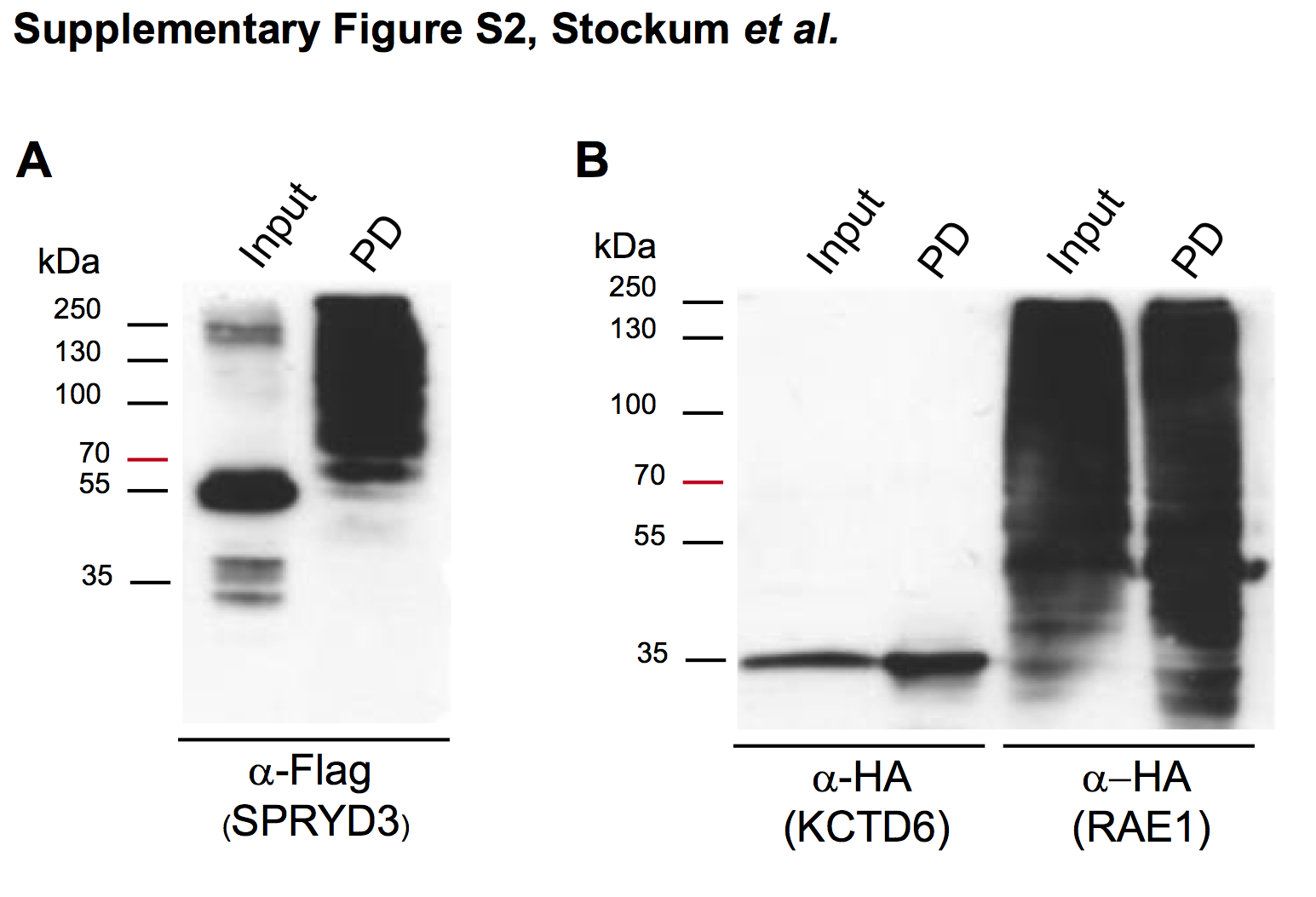

Supplement: S2 Fig — NiNTA pull-down of His6-ubiquitinated proteins under denaturing conditions [28]. 293T cells were transfected with pMT107 and (A) Flag-SPRYD3, (B) HA-KCTD6 or HA-RAE1. Antibodies used are indicated underneath each western blot. (TIFF) [file pone.0190513.s003.tiff]

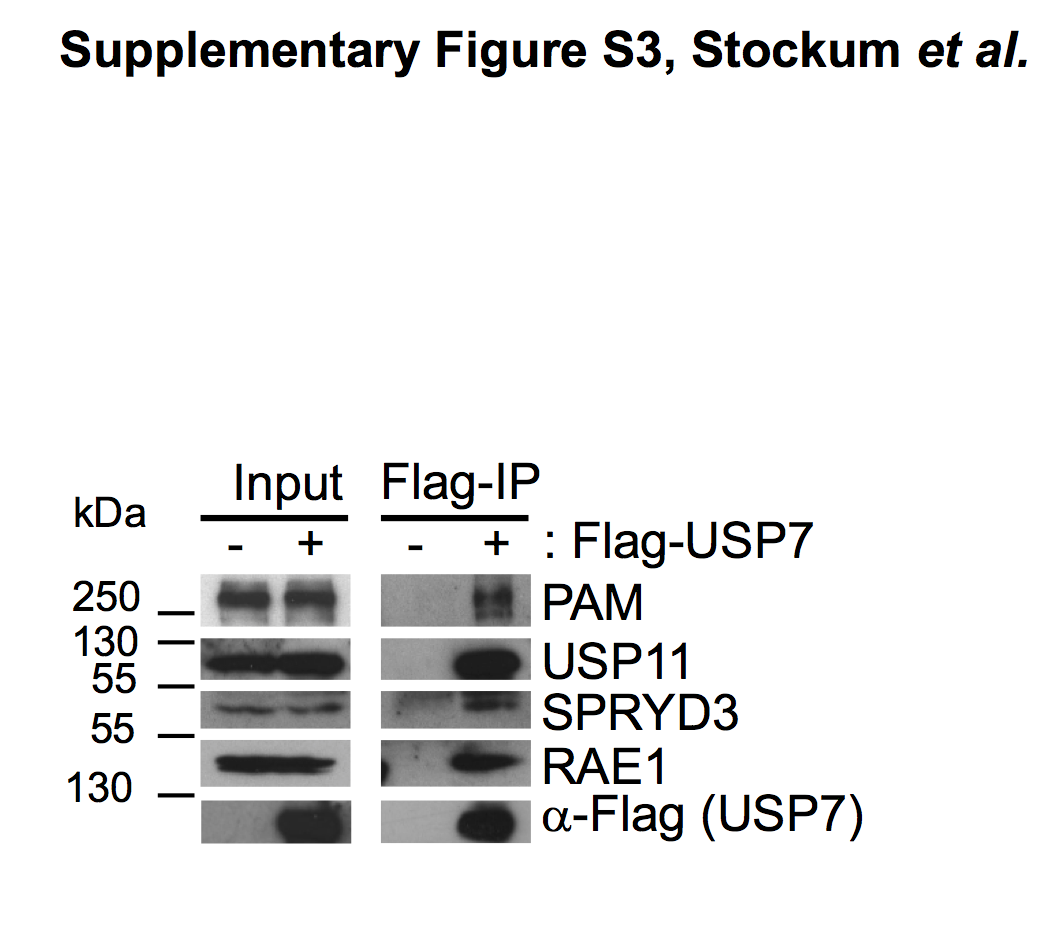

Supplement: S3 Fig — Detection of endogenous proteins are indicated to the right of each western blot. Input and IP are indicated above the western blots. The negative control sample comes from 293T cells transfected with the empty pQFlag-puroR plasmid. 1%, respectively, 10% of the input and IP samples were separated on gel. (TIFF) [file pone.0190513.s004.tiff]

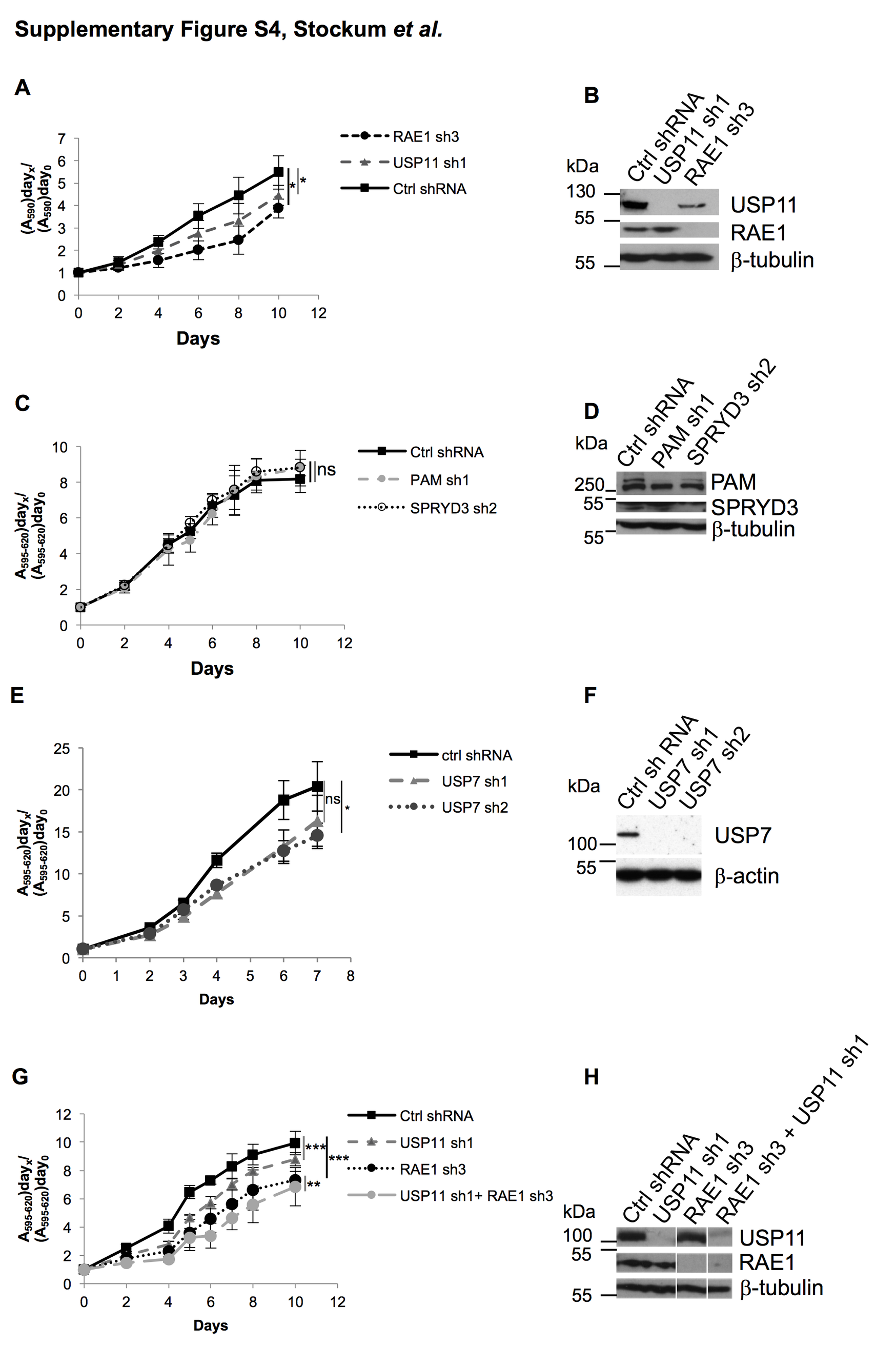

Supplement: S4 Fig — (A) Growth curve of U2OS cells transduced with control, USP11sh1 or RAE1sh3 RNA measured by crystal violet staining. The knock-down of RAE1 or USP11 results in a significant growth defect compared to the control shRNA transduced cells (p = 0.0167; p = 0.042, respectively). (C) Ablation of PAM or SPRYD3 does not significantly change the proliferation of U2OS cells as measured by MTT assay (p = 0.8575; p = 0.05, respectively). (E) Cell viability measured by MTT assay. Knock-down of USP7 reduces cell proliferation compared to control shRNA transduced cells (p = 0.0285 for USP7sh1; p = 0.0547 for USP7 sh2). (G) Cell proliferation measured for U2OS cells transduced with control, USP11sh1, RAE1sh3 or USP11sh1+RAE1sh3 shRNAs as indicated in the legend. No synergistic growth effect was observed by simultaneous ablation of USP11 and RAE1. The cells did, however, grow significantly slower than cells with ablation of RAE1 only (p = 0.0019). Averages and SEM of three independent transductions and growth curves are shown. p-values were calculated using the two-tailed paired t-test, compared to the control shRNA transduced cells, and are indicated as follows: ns: p > 0.05; *: 0.01< p < 0.05; **: 0.001 < p < 0.01; ***: p < 0.001. (B, D, F, H) Western blots illustrating respective protein knock-downs. For each of the panels shown, samples were analyzed on the same western blot. Where a white line is shown, this is to indicate that some lanes, irrelevant to the experiment shown, were removed from the Figure. Antibodies used are indicated to the right of each panel. (TIF) [file pone.0190513.s005.tif]

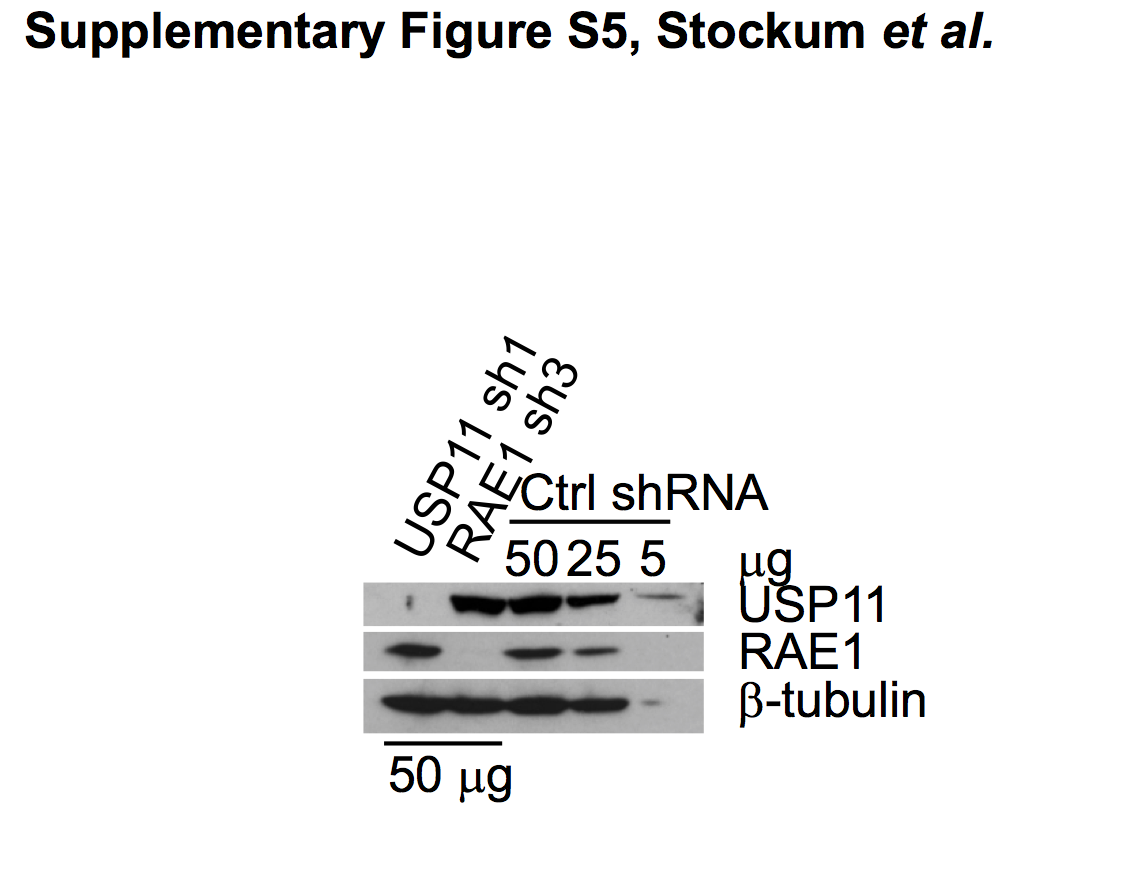

Supplement: S5 Fig — Semi-quantitative western blot analysis of RAE1 and USP11 protein levels illustrates that RAE1sh3 reduces RAE1 protein levels in U2OS cells ~ 10 fold, whilst USP11 protein levels are reduced more than 10 fold. Titration of control shRNA transduced cells as indicated above the western blot. Antibodies used are shown at the right of the western blots. 50 μg of total protein extract was loaded for the USP11 sh1 and RAE1 sh3 transduced cells. (TIFF) [file pone.0190513.s006.tiff]

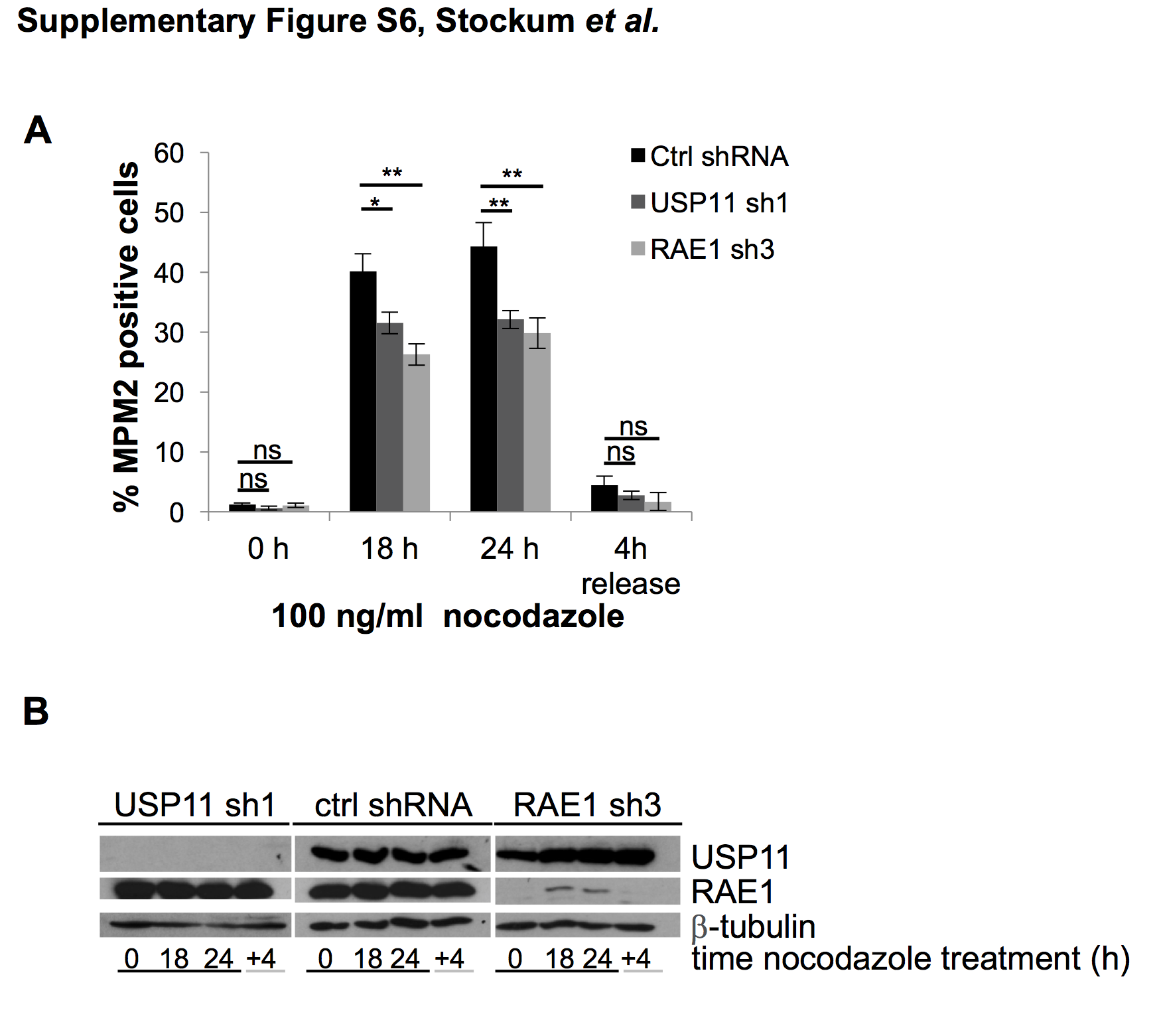

Supplement: S6 Fig — (A) U2OS cells transduced with the indicated shRNAs were arrested with 100 ng/ml nocodazole (or DMSO as negative control “0h”). Cells were harvested 18h or 24h post-treatment and fixed with 70% ethanol, or extensively washed in PBS and released into preheated complete medium following 24h nocodazole treatment (“4h release”). The mitotic index was determined by FACS analysis using MPM2 staining as an indicator of mitotic cells. Less mitotic U2OS cells were measured upon knock-down of USP11 or RAE1 after 18h (p = 0.0126; p = 0.0022, respectively) and 24h nocodazole treatment (p = 0.008; p = 0.0063, for USP11 and RAE1, respectively) in comparison to the control shRNA transduced cells. (B) Western blot analysis of protein levels during nocodazole arrest and after 4h recovery (grey line). All samples were analyzed on the same blot, and are separated here by a white line for clarity. Antibodies used are indicated to the right of the western blots. (TIFF) [file pone.0190513.s007.tiff]

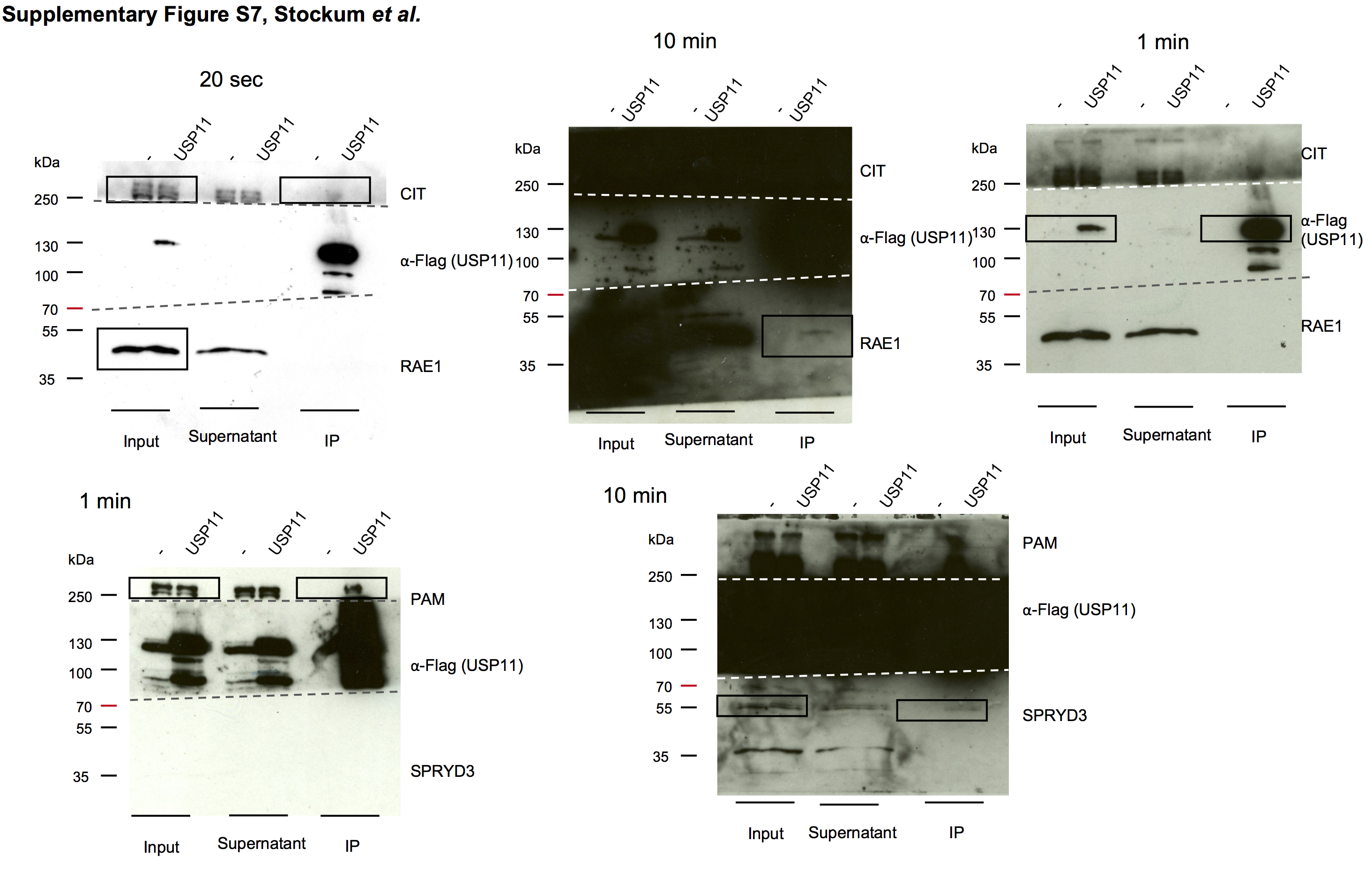

Supplement: S7 Fig — Flag-USP11 IP. Samples were loaded twice and probed with different antibodies. Upper 3 images are different exposure times of the same western blot; bottom 2 are 2 different exposure times of the same western blot. The grey/white striped lines indicate where blots were cut before antibody probing. Antibodies used are indicated to the right of the blots, molecular weight markers to the left. Exposure times are indicated above the western blot. The boxed areas on the western blots indicate which exposures were shown in Fig 1F. (TIFF) [file pone.0190513.s008.tiff]
